# Two structural switches in HIV-1 capsid regulate capsid curvature and host factor binding

**DOI:** 10.1073/pnas.2220557120

**Published:** 2023-04-11

**Authors:** James C. V. Stacey, Aaron Tan, John M. Lu, Leo C. James, Robert A. Dick, John A. G. Briggs

**Affiliations:** ^a^Department of Cell and Virus Structure, Max Planck Institute of Biochemistry, Martinsried 82512, Germany; ^b^Structural Studies Division, MRC Laboratory of Molecular Biology, Cambridge CB2 0QU, United Kingdom; ^c^PNAC Division, MRC Laboratory of Molecular Biology, Cambridge CB2 0QU, United Kingdom; ^d^Department of Molecular Biology and Genetics, Cornell University, Ithaca NY 14853

**Keywords:** retrovirus structure, HIV-1, cryo-electron microscopy, capsid, virus-host interactions

## Abstract

The mature HIV-1 capsid protects the viral genome and interacts with host proteins to travel from the cell periphery into the nucleus. To achieve this, the capsid protein, CA, constructs conical capsids from a lattice of hexamers and pentamers, and engages in and then relinquishes multiple interactions with cellular proteins in an orchestrated fashion. Cellular host factors including Nup153, CPSF6, and Sec24C engage the same pocket within CA hexamers. How CA assembles pentamers and hexamers of different curvatures, how CA oligomerization states or curvature might modulate host–protein interactions, and how binding of multiple cofactors to a single site is coordinated, all remain to be elucidated. Here, using single-particle cryoEM, we have determined the structure of the mature HIV-1 CA pentamer and hexamer from conical CA-IP_6_ polyhedra to ~3 Å resolution. We also determined structures of hexamers in the context of multiple lattice curvatures and number of pentamer contacts. Comparison of these structures, bound or not to host protein peptides, revealed two structural switches within HIV-1 CA that modulate peptide binding according to CA lattice curvature and whether CA is hexameric or pentameric. These observations suggest that the conical HIV-1 capsid has different host–protein binding properties at different positions on its surface, which may facilitate cell entry and represent an evolutionary advantage of conical morphology.

## Significance

HIV-1 particles contain a characteristic, conical capsid that shields the genome from the cellular immune system and recruits cellular proteins to direct the capsid to the nucleus. The cone forms from hexamers of CA protein, and 12 pentamers that accommodate curvature. We obtained detailed 3D models of pentamers and hexamers at positions on capsid surfaces with different curvatures. We find two places in CA that switch conformation according to the local capsid curvature and whether CA is in a pentamer or hexamer. We also obtained models of CA bound to peptides from cellular proteins. The data show how switches in CA help it form a cone shape, and interact differently with cellular proteins at different positions on the cone surface.

The mature HIV-1 capsid is a conical, fullerene shell that encompasses the viral genome. It is constructed of a lattice of approximately 200 capsid protein (CA) hexamers incorporating CA pentamers at 12 highly curved vertices (1, 2). The capsid serves multiple functions during viral infection. As the vessel in which reverse transcription takes place, it regulates access of cellular dNTPs (3) while simultaneously shielding its genetic cargo from detection and degradation by host cellular immunity systems (4–6). The surface of the conical capsid is dense in protein-binding sites that mediate interactions with host machinery necessary for transport of the capsid through the cytoplasm into the nucleus (7–9).

CA monomers consist of two alpha-helical domains. The CA N-terminal domain (CA_NTD_), situated on the outer surface of the capsid, stabilizes the hexamer via interactions around a sixfold axis. The C-terminal domain (CA_CTD_) engages in the dimeric and trimeric interactions that join hexamers together into a lattice. The small molecule inositol hexakisphosphate (IP_6_) is known to act as an assembly factor for both immature and mature HIV-1 CA lattices (10, 11). In the mature CA hexamer, IP_6_ coordinates R18 in the central CA_NTD_ pore (12), where it greatly increases capsid stability (13). Below this, a secondary IP_6_-binding site is formed by a ring of lysines at position K25 (12, 14). The precise stability, or instability, of the capsid is critical for correct function. Accordingly, mutations that hyperstabilize or destabilize the capsid lead to marked reductions in infectivity (15), implying that the capsid must retain sufficient stability to maintain a protective function, but also uncoat readily enough at the correct moment. Flexibility in the linker between the two CA domains and in the interfaces between domains have been proposed to provide plasticity necessary to allow the hexamer to construct different lattice curvatures at different positions on the conical surface (16, 17) and may also permit formation of both pentamers and hexamers of CA. Significant flexibility of the CA_CTD_-CA_CTD_ dimerization interface has been observed in solution and in in vitro assembled tubular arrays of CA (17, 18). In addition, mutation of residues at the hydrophobic threefold symmetry axis can promote pentamer incorporation in vitro, implicating this interface in modulating capsid stability (17). Together the existing data imply that multiple regions of the CA surface are involved in regulating capsid assembly, morphology, and function.

Low-resolution structures of CA hexamers from capsid cores within HIV-1 virions (1) have validated that high-resolution crystal structures of HIV-1 CA hexamers (19) are representative of hexamers in the virion. In contrast, low-resolution structures of the CA pentamer within virions (1) are not consistent with available crystal structures of crosslinked, mutant CA pentamers (20), and a higher resolution structure of the true “in virus” pentamer structure remains to be elucidated.

Two protein-binding sites on the surface of the core have been characterized structurally by way of cryo-EM and X-ray crystallography. The CypA-binding loop constitutes a flexible span of residues between helices 4 and 5, and binds cyclophilin A (CypA) (21) and nuclear pore component Nup358, which contains a CypA domain (22, 23). A second binding site within the CA_NTD_ specifically binds phenylalanine–glycine (FG) motifs (24–26). FG repeat–containing host factors are diverse in their cellular distribution and function (27) and include: Nup153, a nuclear pore complex component; Sec24C, a member of the COPII complex implicated in intracellular vesicle trafficking; and the nuclear-localized CPSF6 (25, 26). All three proteins insert an FG motif into a pocket between helices 3 and 4 of the CA_NTD_, while residues proximal to the FG repeat bind to CA residues in a cleft formed between the CA_NTD_ and the CA_CTD_ of the neighboring CA in the hexamer. Subtle differences in binding affinity between different FG repeats as a result of varying binding conformation may play an important role in transport and nuclear import of the capsid. Accordingly, quantitative fluorescence microscopy of reverse transcription and preintegration complexes suggest that there is a handoff from Nup153 at the nuclear pore to CPSF6 within the nucleus (28). Owing to its critical role in orchestrating early phase replication, the FG-binding site has been the target of concerted efforts to identify CA-targeting antiretrovirals. Such studies have led to potent drug candidates including PF-3540074 (PF74) (29) and members of the GS-CA family including lenacapavir (30, 31). A difference in the relative orientations of the CA_NTD_ and the CA_CTD_ between hexamers and pentamers in in situ structures (1) alters the apparent accessibility of the FG repeat binding site, but in the absence of higher resolution structures of the viral CA pentamer, the implications for host–protein binding to the pentamer are not clear. It is also not clear whether changes in CA hexamer curvature can modulate FG-motif binding.

CA can be induced to assemble in vitro in buffers containing IP_6_ at physiological salt concentration to form structures containing hexamers and pentamers that closely resemble authentic CA cores (32, 33). Here, we present structures at 2.6 to 3.5 Å resolution of CA hexamers and pentamers within in vitro assembled conical CA-IP_6_ polyhedra from multiple lattice contexts differing in local curvature and geometry, alone or in the presence of Nup153-, CPSF6-, or Sec24C-derived peptides. This analysis suggests that the structure and morphology of the core have evolved to preferentially bind different components of the host-cell machinery at different regions of the conical capsid surface. This is achieved using two structural switches within CA that modulate host factor binding. The first switch alters the FG repeat binding pocket between the hexamer and pentamer, precluding FG repeat binding to the pentamer completely. The second switch regulates Nup153 affinity to the hexamer in a manner that is sensitive to local capsid curvature.

## Results

### Structures of the Mature HIV-1 CA Pentamer and Hexamer.

Purified recombinant CA protein was assembled in the presence of IP_6_ into mature core-like particles (CLPs), as described previously (32, 33) (*SI Appendix*, Fig. S1). The contents of the reaction mixture were used to prepare cryo-EM grids, which were imaged in the electron microscope using standard single-particle data collection conditions (*Materials and Methods* and *SI Appendix*, Table S1). Inspection of micrographs confirmed that the size and morphology of the CLPs were qualitatively similar to capsid morphologies reported in mature viruses by cryoelectron tomography (cryo-ET) (1), and this was confirmed using cryo-ET (33).

**Fig. 1.**
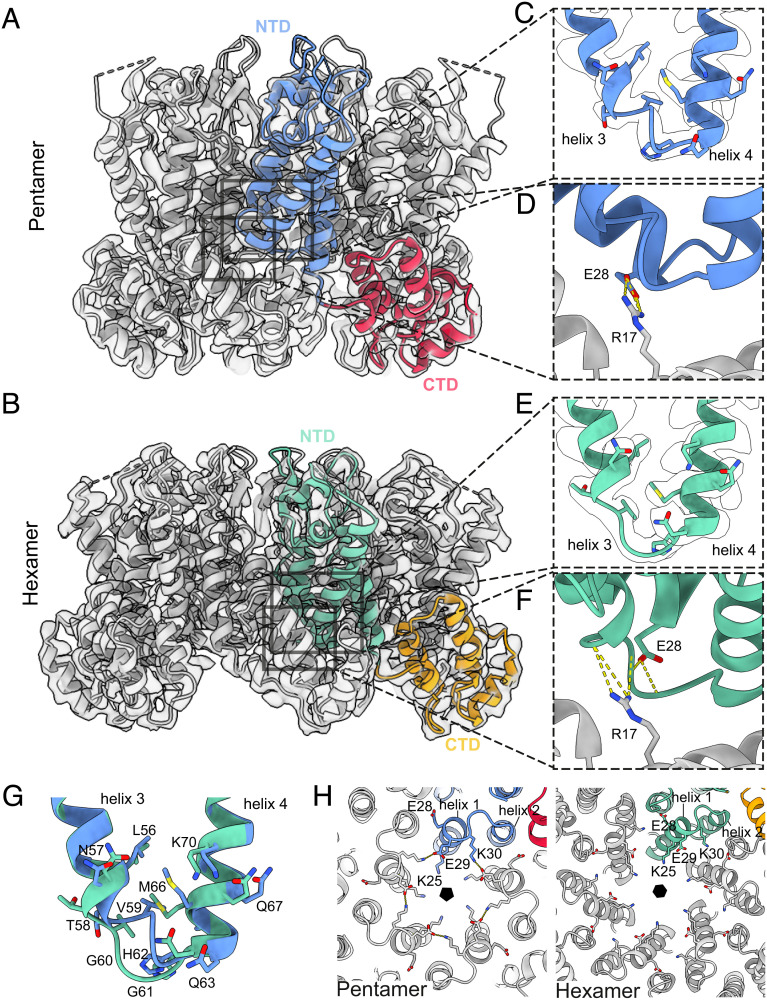
Structure of the HIV-1 CA hexamer and pentamer from in-vitro assembled core-like particles. (*A*) Isosurface representation of a single-particle reconstruction of the CA pentamer, viewed from the side. The corresponding atomic model is shown as ribbons in gray, with one monomer colored in blue (NTD) and red (CTD). (*B*) As in (*A*), for the hexamer reconstruction from the same data. A single monomer is colored in green (NTD) and orange (CTD). (*C*) Zoomed in view of the base of helix 3/4 and the intervening loop (FG repeat binding site) from the pentamer reconstruction and model. (*D*) Zoomed in view of R173, which reaches past the helix 3/4 loop to interact with E28. (E) Zoomed in view of the equivalent region of (C), for the hexamer reconstruction and model. (F) Zoomed in view of the equivalent region of (*D*), for the hexamer reconstruction and model. R173 and E28 are separated by the helix 3/4 loop, with which they interact. (*G*) Superposition of models from (*C*) and (*E*) reveals structural differences between the pentamer and hexamer at the FG-binding site. As compared to the hexamer (green), the pentamer (blue) has a 3_10_ helical turn at the base of helix 3, V59 is located further toward the center of the binding pocket and M66 adopts a different conformation. We refer to this region as the “hexamer–pentamer switch.” (*H*) View of the central CA_NTD_ pore, from inside the CLP, showing an exchange of binding partners between hexamer and pentamer for charged residues.

From these data, we selected “particles” corresponding to arbitrary regions of the capsid surface and applied single-particle data analysis techniques including 3D-classification (*Materials and Methods*) (*SI Appendix*, Fig. S1). We obtained separate reconstructions of the pentamer and of the hexamer to nominal resolutions of 3.1 Å and 2.9 Å, respectively, and used these to build atomic models (Fig. 1 *A* and *B* and *SI Appendix*, Fig. S5). Both structures match well to those previously determined at low resolution within virions (*SI Appendix*, Fig. S6), and to those independently determined in the accompanying manuscript (33).

The relative orientation of the CA_NTD_ and CA_CTD_ within individual CA monomers is very similar in hexamers and pentamers, with very little change in the position of the interdomain linker. Comparison of the pentamer and hexamer CA_NTD_ structures revealed structural differences at the base of helices 3 and 4 and the intervening helix 3/4 loop, and includes the formation of an additional 3_10_ helical turn at the base of helix 3 (residues 58 to 61) in the pentamer (Fig. 1 *C*–*G* ). This results in V59 moving toward the core of the CA_NTD_ helical bundle, inducing a different conformation of M66. This restructures the pocket which, in the hexamer, is the binding site for FG repeat cofactors Nup153, CPSF6, and Sec24C. Based on comparison with crystal structures of FG repeat peptides bound to CA hexamers, we speculated that in the pentamer this pocket is unable to bind FG motifs. We will refer to this structural rearrangement as the “hexamer–pentamer switch.” These observations match and confirm those made in a recent study from the Pornillos lab (34).

In the hexamer, as in the crystal structure, interactions between neighboring CA_NTD_s around the symmetry axis are mediated in large part by residues P38 and M39 in helix 2 contacting residues N57 and T58 in helix 3 from the adjacent monomer. In contrast, in the pentamer, helix 2 forms an interface with helix 1 of the adjacent monomer (Fig. 1*H*). Additionally, a new interface forms between helix 1 of adjacent monomers, including a hydrogen bond between T19 and the εN of R18 that may further fix the position of the R18 ring, and a salt bridge between K30 and E29. In the hexamer, K30 is not a pore-facing residue and may interact with E28, while E29 is freely exposed to the centre of the pore (Fig. 1*H*).

Within the central pore of the pentamer, we observe two densities, which we interpret as IP_6_ molecules (33): one IP_6_ sits above, and engages with, the R18 ring, while the other is coordinated by the K25 ring in a fashion that is reminiscent of previously reported hexameric crystal structures (13) (*SI Appendix*, Fig. S6). Within the central pore of the hexamer, we also observe two IP_6_ densities coordinated by rings of R18 and K25, but density for IP_6_ coordinated by K25 appears to be weaker than that of the pentamer suggesting lower occupancy. The spacing between K25 residues is much larger in the hexamer, and its position suggests that in the absence of IP_6_, it could instead form an intramolecular salt bridge with the exposed E29.

The CA_NTD_–CA_CTD_ interface in the hexamer is essentially identical to that previously observed in both pentameric and hexameric CA crystals [PDB: 4XFX, PDB:3P05; (19, 20)]. In contrast, in our pentamer structure, the CA_CTD_ forms an interface with a position markedly lower on the neighboring CA_NTD_ (*SI Appendix*, Fig. S7). Here, a largely hydrophobic interface forms centered around Y169, L211, and M215 in the CA_CTD_, and A64, M144, and Y145 in the CA_NTD_. R173 in the CA_CTD_, which in the hexamer forms a hydrogen bond with the backbone of hexamer–pentamer switch residues N57 and V59 in the adjacent CA_NTD_ (Fig. 1*F*), instead interacts with E28 in the pentamer (Fig. 1*D*).

### Structures of Hexamers of Varying Curvature.

**Fig. 2.**
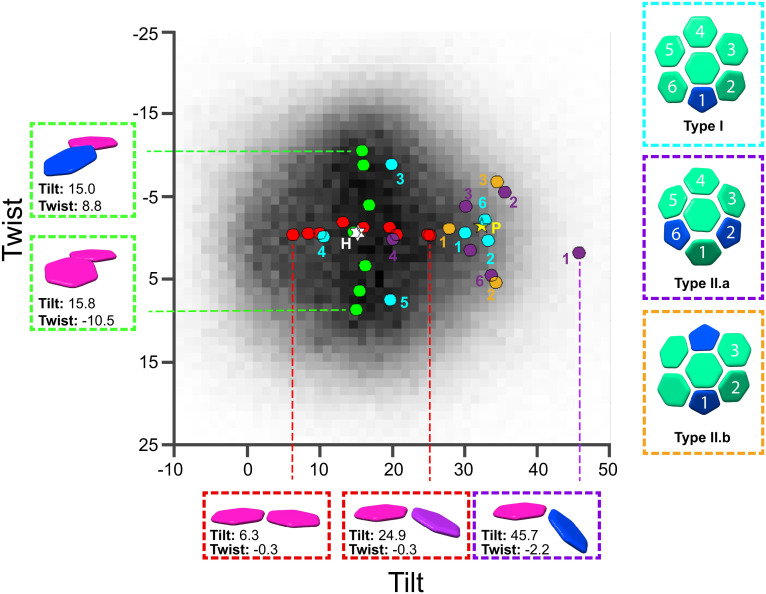
Curvature variation across the mature HIV-1 CA-IP_6_ cores. Heat map of measured tilt and twist angles for all hexamer–hexamer pairs identified in tomograms. Colored circles on the heatmap represent the tilt and twist angles of hexamer–hexamer and hexamer–pentamer pairs resolved in single-particle reconstructions of individual classes. White star “H”: the hexamer–hexamer orientation in the average C6 structure from all hexamer data. Yellow star “P”: the pentamer–hexamer orientation in the average C5 structure from all pentamer data. Red: class averages from particles grouped according to tilt. Green: class averages from particles grouped according to twist. Numbered circles indicate the orientation from the central hexamer to the adjacent oligomer for hexamers that are immediately adjacent to pentamers, as numbered in the side panels for geometry type I (cyan), type II.a (purple), type II.b (orange) – see text for further details.

To form the surface of the conical HIV-1 capsid, hexamers adapt to different local curvatures at different positions of the core surface, and in certain instances make contact with one or more pentamers. In order to study curvature and contact variation in our sample, and following the approach previously applied to cores within intact HIV-1 virions (1), we first analyzed cryo-ET/subtomogram averaging data of the assembled CLPs (33), and calculated tilt and twist angles between all pairs of neighboring hexamers. The distribution of our tilt–twist measurements represents a distribution of curvatures in the CLPs (Fig. 2, heatmap) and recapitulates the previous observations made in virions (1). Additionally, through visual inspection of the hexamer–pentamer distributions revealed by cryoET, we identified 4 classes of hexamer that make contact with a pentamer: hexamers contacting one (type I), two (2 forms, type II.a and II.b) (Fig. 2), and, in rare cases, three pentamers (type III). All of these classes have also been observed within viruses (see *SI Appendix*, Fig. S7 in ref. 1).

To study the structures of hexamer curvature variants within our single-particle dataset, we applied symmetry expansion to generate a dataset corresponding to all hexamer–hexamer pairs, and then performed 3D variability analysis. From this analysis, we derived two primary variability components, which upon inspection resembled hexamer–hexamer tilt in the first class and hexamer–hexamer twist in the second class. We grouped the refined particles along these two variability components to generate nine nonoverlapping classes for tilt and seven for twist, each of which was independently refined and reconstructed. Doing so allowed us to generate two series of reconstructions varying by tilt and twist, where all individual reconstructions were at resolutions between 3.1 and 3.4 Å. The tilt and twist values for each reconstruction were measured from the symmetry axes of fitted models and mapped onto the distribution determined by cryo-ET (Fig. 2, red and green points). Doing so confirmed that the two primary variability components correspond cleanly to tilting and twisting and that the angular ranges represented in our reconstructions (Tilt: +6.3° - +24.9°, Twist: −10.5° - +8.8°) constitute a significant portion of the true accessible range (Movies S1 and S2).

Next, using reference-based classification, we identified classes of hexamer that were adjacent to pentamers within our original hexamer particle set, which we then independently refined. These yielded reconstructions of hexamers contacting a single pentamer, resolved to 3.0 Å and two reconstructions of hexamers contacting two pentamers (type II.a and II.b), resolved to 3.6 Å and 3.9 Å, respectively. We were unable to recover a class corresponding to hexamers contacting three pentamers, likely due to its rarity within our sample. To describe the geometry of hexamers adjacent to pentamers, we calculated the tilt and twist of all the resolved hexamer–pentamer and hexamer–hexamer pairs. Hexamer–hexamer pairs adjacent to pentamers are highly tilted (Fig. 2, black, gray, and white points): For example, the two symmetry-related hexamers in the type II.a reconstruction are tilted relative to one another by 45.7°.

### Interpretation of Hexamer Variant Structures and Interfaces.

**Fig. 3.**
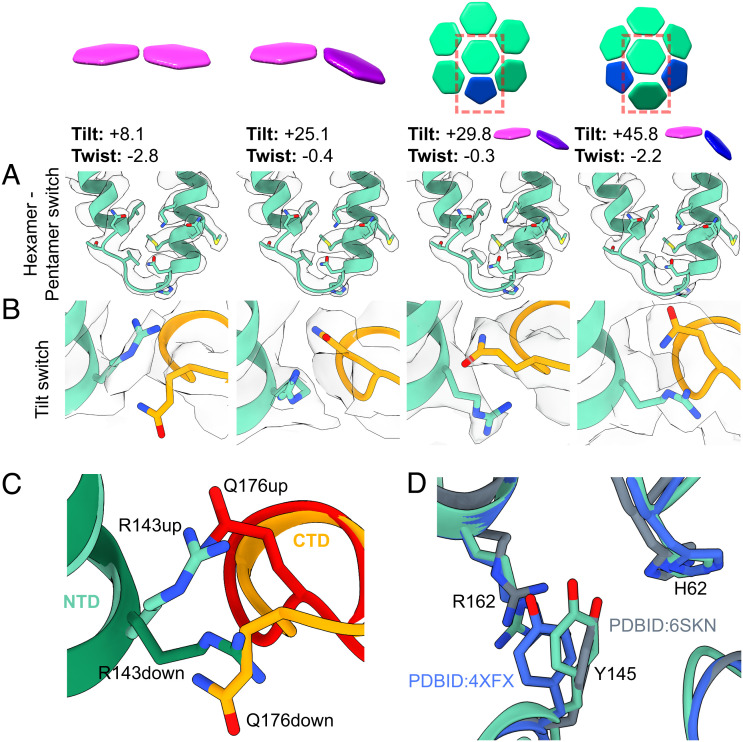
Structures of hexamer tilt variants. (*A*) Isosurface and fit models (green) of the hexamer–pentamer switch region across hexamer tilt variants. The structure of the switch is independent of tilt or twist. (*B*) Isosurface and fit models of the tilt-switch region across hexamer tilt variants. At low tilts, Q176 is below R143, whereas at high tilts Q176 is above R143. At high tilts, there is a shift in the backbone in the vicinity of residue Q176, which is not observed in CA monomers forming hexamer–pentamer contacts. (*C*) Superposition of models of the tilt-switch region from CA monomers in low tilt regions (light green (NTD) and light orange (CTD)) and high tilt regions (dark green (NTD) and dark orange (CTD)). (*D*) Superposition of the average hexamer model (green), with PDBID:6SKN (16) (gray) and PDBID:4XFX (19) (blue), showing the relative configurations of H62 and Y145 as well as R162 of the neighboring monomer.

The above approach generated 19 reconstructions representing the curvature variability of capsid on the surface of the CLPs. We built models into these reconstructions and compared them with one another. All hexamers, regardless of tilt or twist, share the same structure in the “pentamer–hexamer switch” region (Fig. 3*A*). From this observation, we conclude that the structural rearrangement of the switch region does not simply represent the most-tilted end of a continuum of increasing lattice curvature, but is a true pentamer–hexamer switch.

We observe curvature-dependent changes at the base of helix 7 in the CA_NTD_ and in the helix 8/9 loop in the CA_CTD_ with which helix 7 forms a small interaction interface (Fig. 3 *B* and *C*). In the average hexamer structure, the R143 sidechain is placed above the helix 8/9 loop, with Q176 pointing downward. This is the conformation observed in all lower tilt structures (and in the average structure shown in Fig. 5*E*). In contrast, at the most highly tilted hexamer–hexamer contacts, including those in the vicinity of pentamers, R143 is positioned below the helix 8/9 loop and Q176 points upward. At these positions, there is also a shift in the backbone at E175-Q176. At medium tilts, we observe a mixture of the two conformations. R143/Q176 do not appear to reconfigure in response to twist. CA molecules that form hexamer–pentamer contacts form a defined intermediate configuration in which Q176 is above R143, but the backbone at E175-Q176 is in the “low-tilt” conformation (Fig. 3*B*, third column). We will refer to these structural rearrangements as the “tilt switch.”

**Fig. 4.**
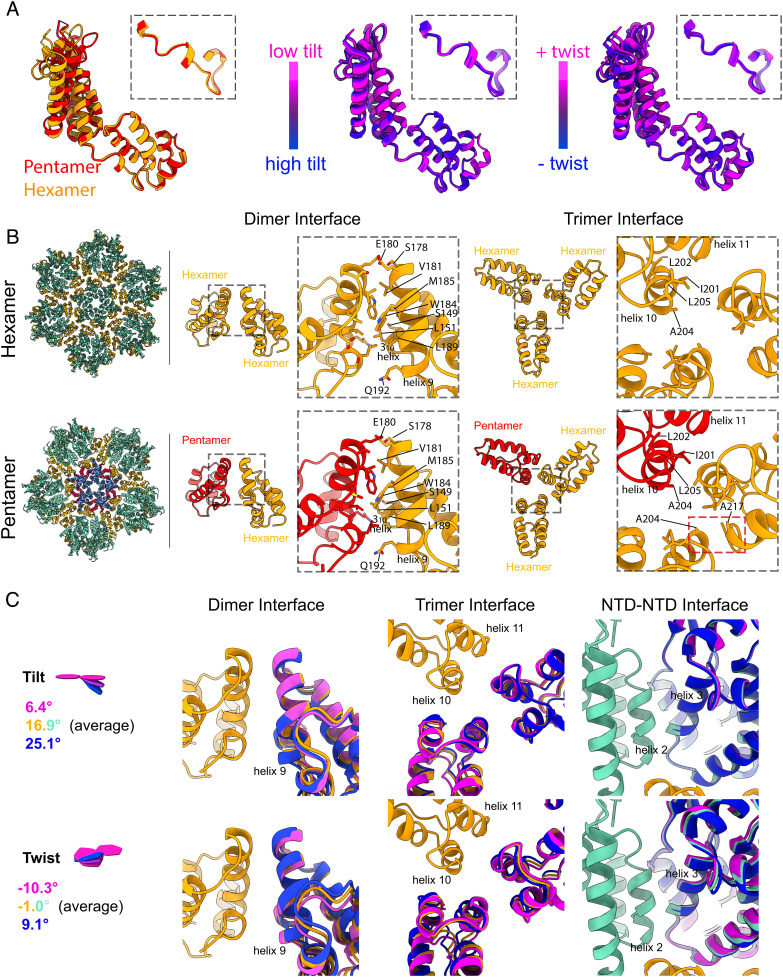
Flexibility of CA and its interactions. (*A*) Superposition of CA structures from the hexamer (orange), and pentamer (red). Superpositions of structures obtained from different tilt/twist classes, colored blue to magenta. Structures are aligned on the CA_CTD_, insets illustrate that there is minimal motion around the interdomain hinge. (*B*) The dimeric interface between CA molecules mediated by helix 9, and the trimeric interface mediated by helix 10, for interfaces involving only hexamers (orange), or also including pentamers (red). The dimeric interface is largely conserved, whereas trimeric contact points that include a pentamer form a new interaction between helices 10 and 11. (*C*) Superposition of structures obtained from classes with large tilt/twist values and the average structure at the dimeric and trimeric interfaces, as well as in the region of helices 2 and 3 in the NTD. Minimal structural changes are observed at the dimeric interface, whereas the trimeric interface show rotations at hydrophobic interfaces. Tilt/twist angles are provided and are colored consistently with models. Dimer and trimer interface structures were aligned to the CTD at the left of the panel; NTD–NTD interface structures were aligned to the NTD on the left of the image. See also *SI Appendix*, Fig. S8.

Previous structures of CA hexamers assembled into helical tubes have suggested that Y145 engages in an intramolecular hydrogen bond with H62 in some positions within highly curved hexamers (PDBID: 6SKN), contrasting with the situation in planar hexamer crystals where Y145 forms an intermolecular contact with R162 (PDBID: 4XFX) (16, 19, 35). In contrast, we observe no curvature dependence of interactions involving Y145, H62, and R162 in our curvature variant structures – in all cases, as in the average hexamer structure, the position of Y145 is intermediate between the two previously observed positions (Fig. 3*D*). Indeed, the entire interdomain hinge, of which Y145 is the N-terminal residue, is very similar in all structures (Fig. 4*A*).

We next analyzed the protein–protein interfaces that mediate hexamer–hexamer interactions at the dimeric and trimeric interfaces in the lattice, as well as at the quasi-equivalent interfaces involving the pentamer. The dimeric interface formed by helix 9 is remarkably invariant across the core surface, and matches the structure and minimal plasticity observed in previous studies (16, 19) (Fig. 4*B* and *SI Appendix*, Fig. S8). This is in contrast to earlier suggestions that variable curvature is accommodated based on plasticity of the dimerization interface (17). The threefold lattice interface formed by helix 10, however, shows variation. At interfaces between three hexamers (independent of tilt and twist angle), helix 10 engages in a symmetrical three-helix bundle, held in place by a hydrophobic patch which includes residues I201, L202, A204, and L205, as previously observed in helical arrays (17). At interfaces between a pentamer and two hexamers, one hexamer helix is removed from the bundle and instead forms a new contact with helix 11 of the other hexamer, involving residues A204 and A217 (Fig. 4*B*). There is a small amount of flexibility in the precise structures of hexamer–hexamer threefold lattice interfaces as a result of tilt and twist variation (Fig. 4*C* and *SI Appendix*, Fig. S8), likely facilitated by the nonspecific nature of the hydrophobic interactions at this site; however, none of these structures resemble the structure at the hexamer–pentamer interface. The hydrophobic helix 2–3 interface around the central pore acts as a pivot to allowing small relative motions of neighboring CA_NTD_ domains in response to lattice bending and twisting (Fig. 4*C*). In summary, subtle but cumulative variations in interhexamer and intrahexamer interfaces are sufficient to accommodate variations in curvature across the hexameric CA lattice, while modulation of the trimer interface at the contact between pentamers and hexamers is required to accommodate the highly curved pentameric geometry.

### Structures from Cores Bound to FG Repeat Peptides.

**Fig. 5.**
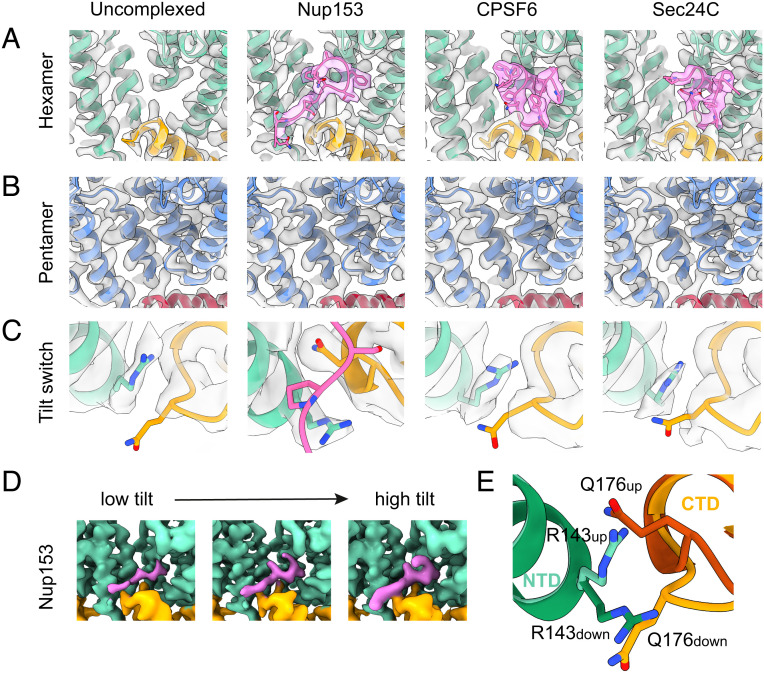
The hexamer–pentamer switch and the tilt switch regulate cofactor binding. (*A*) Isosurface representation and ribbon models of the FG-binding pocket of the CA hexamer reconstructed from cores incubated without peptide and with peptides from Nup153_(1407–1423)_, CPSF6_(313–327)_ and Sec24C_(228–242)_. The NTD is colored green, the CTD is orange, and peptide is pink. (*B*) The equivalent pocket in the pentamer is empty in all four cases. (*C*) Zoomed in view of the tilt-switch region of the hexamer reconstructions for each of the four samples, colored as in (*A*). The Nup153_(228–242)_ bound reconstruction has a tilt-switch conformation the same as observed in highly tilted uncomplexed samples. (*D*) Isosurface volumes of Nup153_(1407–1423)_ bound CA monomers engaging in low (+6.3°), medium (+16.0°), and high-tilt (+24.9°) lattice interactions, colored as in (*A*). Nup153_(1407–1423)_ occupancy increases at higher tilt angles. (*E*) Superposition of the tilt-switch region from the uncomplexed hexamer (light colors) and the Nup153_(1407–1423)_-bound hexamer (dark colors).

To investigate what effect the hexamer–pentamer switch and the tilt switch have on host–protein binding, we next repeated our structural analysis on cores incubated with FG repeat containing peptides Nup153(1407-1429), CPSF6(276-290), and Sec24C(228-242). All three peptides have been described to bind to mature CA hexamers via an FG motif (25, 26). We determined structures of hexamers from peptide-bound cores to nominal resolutions of 2.6 Å, 3.1 Å, and 3.1 Å and associated pentamers to 3.1 Å, 3.5 Å, and 3.2 Å, respectively (*SI Appendix*, Fig. S5). Within all three hexamer reconstructions, we observe unambiguous density for the corresponding peptides (Fig. 5*A*), which adopt structures essentially identical to previously reported crystal structures (24–26). In contrast, we do not detect any density corresponding to peptides bound to pentamers (Fig. 5*B*), though in all three cases, peptide density is visible in the binding pocket of the hexamers immediately adjacent to the pentamers (*SI Appendix*, Fig. S9). From these observations, we conclude that the hexamer–pentamer switch described above prevents binding of the FG motif in these peptides to the pentamer. Superposition of our peptide-bound hexamer CA_NTD_ structures and our pentamer CA_NTD_ structure confirms that residue M66 in the pentamer would clash with the binding position of phenylalanine within the pocket (*SI Appendix*, Fig. S10).

Further comparison of our peptide bound and unbound hexamer structures revealed that the tilt switch adopts the bulk, lower tilt conformation in the average hexamer in the CPSF6(276-290)- and Sec24C(228-242)-bound capsids. This is the same conformation as seen in the average unbound apo hexamer. In contrast, in the average Nup153(1407-1429)-bound capsid, the switch is in the high-tilt conformation (Fig. 5*E*) with R143 positioned below the helix 8/9 loop, Q176 pointing upward and the shift in the backbone position at E175-Q176. The peptide-bound structures are, in all cases, consistent with the available crystal structures, suggesting that the interaction of P1411 and S1412 in Nup153 with Q176, A177, and R143 in CA stabilizes the high-tilt conformation of the tilt-switch (Fig. 5*C*). The FG motifs in Sec24C and CPSF6 adopt a more compact structure and do not interact with these CA residues. We proceeded to classify the Nup153(1407-1429) in bound hexamers according to tilt/twist and position relative to pentamers to generate context-specific structures exactly as described above for the apo capsid. This analysis yielded structures at resolutions from 3.1 to 3.7 Å. Inspection of these resulting densities revealed that the Nup153-bound hexamers adopt the high-tilt conformation at all tilt angles, suggesting that Nup153 binding stabilizes the switch in the high-tilt conformation even at low tilt angles. We did however, detect an increase in relative Nup153 peptide density at higher tilt angles, suggesting increased peptide occupancy (Fig. 5*D*). We posit that the increase in observed Nup153(1407-1429) occupancy at higher tilts correlates with the arrangement of the R143/Q176 tilt switch in the apo form, because the high-tilt conformation is favorable for Nup153(1407-1429) binding.

## Discussion

CA must be flexible enough to accommodate curvature differences across the surface of the conical core, where angles between neighboring hexamers can vary by 30°. It must also be flexible enough to fill both pentameric and hexameric positions in the core surface. The scenario once considered most likely was that flexibility was accommodated by the interdomain hinge and the dimeric interface involving helix 9, both of which vary in solution and in helical arrays of CA (16, 17). Our previous low-resolution analysis of the CA flexibility within intact virions suggested that, instead, flexibility is accommodated by small structural changes distributed throughout CA (1), and this is largely consistent with recent analysis of helical CA arrays (16). The higher resolution data presented here allow structural changes to be analyzed at the amino acid level. Both the interdomain hinge and the dimeric interface are homogeneous across the full range of hexamer–hexamer curvatures, and at interfaces between hexamers and pentamers. As suggested by the low-resolution analysis, variable curvature is indeed accommodated by small, distributed changes that do not impact the local bonding interactions between amino acids, but that can combine over longer distances to change curvature. Hydrophobic interfaces at the threefold axis and in the helix 2 to 3 interface around the central hexamer pore provide slippery surfaces that can help accommodate flexibility of the hexamer. Within the hexameric lattice, the only clear exception to this general observation is the tilt switch.

How does CA adapt its structure at the pentameric positions in the lattice? The twofold interface at helix 9 formed by the pentamer-forming CA_CTD_ with the neighboring hexamer is essentially the same as the interface between two hexamers, while the hydrophobic interfaces at helix 10/11 adapt to the change in geometry at the pseudothreefold axis between one pentamer and two hexamers. The relative positioning of the CA_NTD_ and CA_CTD_ within the monomer is also very similar between pentamers and hexamers. Indeed, superimposing the CA_CTD_ of a monomer from the hexamer, with that of a CA monomer from the pentamer, results in a closely overlapping CA_NTD_ with minimal difference in the hinge orientation (*SI Appendix*, Fig. S11). Overall, the impression is that the CA lattice around the fivefold position continues to grow via a conserved twofold interface into the fivefold position. This growth positions the CA_NTD_ relative to the neighboring CA_CTD_ such that it would lead to a clash of the CA_NTD_ loop between helices 3 and 4, and R173 in the neighboring CA_CTD_ (*SI Appendix*, Fig. S11). This clash is resolved by the “hexamer–pentamer switch” which alters the position of this loop, allowing interaction between E28 and R173 to stabilize the pentamer-specific packing of CA_NTD_ and neighboring CA_CTD_ (Fig. 1*D*). This may lead to an exchange of interactions: In the pentamer E29 interacts with K30, while in the hexamer E28 may interact with K30, leaving E29 exposed in the pore to possibly interact with K25 in the absence of IP_6_ (Fig. 1*G*). The E28A/E29A double mutant, as well as the R173K mutant, are able to assemble and release immature-like particles but are noninfectious (36, 37). The pentameric packing results in a much more compact arrangement of helix 1 around the pore and a much greater charge density where the K25 residues from five CA molecules are close together. This provides a possible explanation for why pentamer formation, in contrast to hexamer formation, is dependent on K25 and IP_6_ or similar charged molecules for assembly (33).

The hexamer–pentamer switch also alters the conformation of the FG repeat binding pocket. Our structures in the presence of host peptides show that this change prevents binding of FG repeat peptides from Nup153, CPSF6, and Sec24C to this pocket in the pentamer. What are the possible implications of this for core function? On one hand, it lowers the density of FG repeat binding sites at the tips of the core, in particular at the highly curved tip of the narrow end of the core where pentamers are enriched. This may facilitate access for proteins that bind elsewhere on CA, for example the cytoplasmic Nup358 may more easily interact with the CypA loop at the tips of the cores if the occupancy of other proteins to the FG repeat binding site is lower. On the other hand, it provides a pentamer-specific structure, that may provide a binding site for as-yet-unknown host proteins that are required for transport to or into the nucleus.

The tilt-switch provides a second mechanism by which host proteins might preferentially bind particular regions of the core surface. It would allow Nup153 to preferentially bind to the more curved regions of the core, which are localized toward the ends of the cone (*SI Appendix*, Fig. S12). Where different cellular proteins are competing for the same pocket in capsid, this might in turn lead to other proteins binding to flatter parts of the capsid surface. What are the possible implications of this for core function? On the one hand, it may allow different host proteins to cluster on different regions of the core surface, creating local functional surfaces with high allostery. On the other hand, it may orient the core in defined ways, for example positioning regions of higher curvature toward regions of high Nup153 density during nuclear entry (38).

We speculate that maintaining local regions of core surface that specialize in interaction with different host proteins is one of the evolutionary advantages provided by the unusual conical shape of the HIV-1 core.

## Materials and Methods

### Sample Preparation.

Proteins were recombinantly expressed in *Escherichia coli* and purified by ammonium sulfate precipitation and ion exchange chromatography, exactly as described in ref. 33. In vitro assembly of CLPs was performed by incubation with IP_6_ exactly as described in ref. 33.

### Cryo-EM Grid Preparation.

C-Flat CF-2/2-3Cu-50 grids were glow-discharged for 45 s with a current of 25 mA in a PELCO easiGlow glow discharger immediately before use. All samples were vitrified using a Thermo Fisher Scientific Vitrobot Mark IV, operated at 100% humidity and 18 °C.

In the case where no peptide was added to the conical CA-IP_6_ cores, the sample mixture was made by mixing CLPs in assembly buffer with BSA-conjugated 10-nm gold fiducials in 1× PBS at a ratio of 8:1. For peptide-binding experiments, an additional peptide-binding step was carried out in order to prepare the sample for plunge-freezing. Conical CA-IP_6_ cores were mixed with a volume of BSA-conjugated 10-nm gold fiducials in 1× PBS, calculated to give a final core:gold ratio of 8:1 after peptide addition. The peptides used were Sec24C residues 228 to 242, CPSF6 residues 313 to 327, and Nup153 residues 1,407 to 1,423 and were obtained either from Donna Mallery (MRC Laboratory of Molecular Biology) (synthesized by Designer Biosciences) (Nup153 and CPSF6) or the Max-Planck Institute for Biochemistry Peptide Service (Sec24C) as a lyophilized powder. A solution of the respective peptide at 10 times the required concentration, in core assembly buffer containing 1% DMSO, was then diluted 1:10 in this mixture of cores and gold fiducials, and incubated on ice for 15 min prior to use.

Four microliters of the sample mixture for plunging was applied to the carbon side of grids within the humidity chamber of the Vitrobot. The sample was then manually blotted from the opposite side of the grid for 3 s using Whatman No. 1 filter paper, and then plunge-frozen in liquid ethane. Grids generated in such a way were compatible with both tomography and single-particle data acquisitions.

### Single-particle data collection.

All data for single-particle analysis were collected on a Titan Krios G3i cryo-Electron TEM (TFS) operated at 300 keV equipped with a Falcon 4 direct electron detector. Movies were collected at a nominal magnification of ×130,000 with a resulting pixel size of 0.93 Å and a total accumulated dose of ~40 e^−^/ Å at under focus values ranging from −0.6 to −3.0 μm in steps of 0.2 μm. Data were collected as movies automatically with EPU acquisition software (TFS). Data collection parameters are summarized in *SI Appendix*, Table S1.

### Image Processing.

Dose-fractionated movies were aligned, dose-weighted, and averaged with MotionCor2 (39) in RELION-4.0 (40). Automated particle picking was performed using crYOLO (41) on motion-corrected micrographs. Initial pick annotations and model training were performed independently for each of the four single-particle datasets. In order to generate an initial annotated training set, a subset of 100 micrographs of each dataset were manually picked in the crYOLO boxmamanger GUI; overlapping picks were placed across the entire visible surfaces of HIV-IP_6_ cores without attempting to define individual hexamers or pentamers, whether such features were visible or not.

Motion corrected micrographs and picked positions were imported into cryoSPARC (42, 43) where initial defocus estimates were calculated using Patch CTF estimation. Using the same initial set of picked particles, either refined hexamer or refined pentamer positions were derived with parallel rounds of heterogeneous refinement using either hexamer or pentamer reconstructions as initial references, both of which were obtained previously from subtomogram averaging (33). Resulting classes from these refinements that did not resemble the targeted structure, or were of visibly low quality, were discarded and particles from selected classes were pooled and used for further refinement.

For structures of hexamers adjacent to pentamers, particle positions were derived in one of two ways; either through 3D-classification of previously derived hexamer positions, as was the case for the uncomplexed and Nup153_(1407-1423)_ datasets, or through symmetry expanding pentamer positions and shifting of the particle box onto the neighboring hexamer positions, as was the case for CPSF6_(313-327)_ and Sec24C_(228-242)_ bound datasets. Further 3D-classification of these particles was employed to detect further type II.a and II.b arrangements.

In all cases, once initial particle positions were identified, nonuniform refinement was performed, followed by local CTF-refinement and then local masked-refinement with updated CTF values. Where an increase in resolution could be gained, additional rounds of heterogeneous refinement followed by local refinement were performed. For the uncomplexed and Nup153_(1407-1423_)-bound datasets, particle positions were imported back into RELION-4.0, using pyem, where particle motions were corrected using Bayesian polishing. Polished particles were then imported back into cryoSPARC where a final round of local refinement was performed.

### Building and Refinement of Atomic Models.

All CA models were derived principally from a crystal structure of full-length HIV-1 CA, PDB: 4XFX (19). Initial coordinates of peptide ligands were sourced from PDB:6PU1 (Sec24C), PDB:5STX (Nup153), and PDB:4U0A (CPSF6). The CA_NTD_(1-147), CA_NTD_(148-230) and peptides were independently docked into their respective density as rigid bodies within UCSF chimera (44). Atomic positions and geometry were refined using ISOLDE as a plugin within UCSF ChimeraX (45, 46). Manual adjustment of side chain rotamers was performed in COOT. For nonsymmetrical hexamers adjacent to pentamers, this was performed for all six CA chains independently. Models were finally refined as complete hexameric or pentameric assemblies using PHENIX.real_space_refinement (47) with noncrystallographic symmetry enforced where appropriate. Validation statistics were calculated using MolProbity (48). A summary of refinement and validation statistics can be found in *SI Appendix*, Table S1.

The above approach was used to generate models for Apo and peptide-bound average pentamers, average hexamers and Type 1 hexamers next to pentamers. For Type 2 hexamers next to pentamers and for tilt and twist classes, domains from monomers from the average structures were fitted as rigid bodies and local regions relevant for interpretation were manually adjusted.

### Tilt/twist Analysis of CLPs.

Cryo-ET data of CLPs were collected using standard procedures and processed by subtomogram averaging to obtain CA hexamer and pentamer structures and the positions and orientations of hexamers. The cryo-ET data and the methods for data collection and processing are described in ref. 33. The tilt and twist distribution (heatmap in Fig. 2) was calculated from subtomogram positions and orientations exactly as described in ref. 1.

### Tilt/Twist Classification of CA-IP_6_ Hexamer–Hexamer from Single-Particle Reconstructions.

In order to derive tilt and twist classes for hexamer–hexamer pairs, our final C6 hexamer reconstruction was subjected to symmetry expansion and then 3D variability analysis in cryoSPARC (49). The mask used for 3D variability analysis consisted of the central hexamer and one neighboring hexamer. Three-dimensional variability components corresponding to tilt and twist could be identified by visual inspection, and consistently were found within the top three reported components. Particles were grouped along the two variability components, nine groupings for tilt and seven for twist. Pooled particles were then subjected to local refinement with no symmetry applied to yield a final reconstruction.

### Measuring Tilt/Twist of Single-Particle Reconstructions.

Previously deposited hexameric (5MCX) or pentameric CA structure (5MCY) (1), models were rigidly fit into adjacent densities using UCSF Chimera. Within each of the two fit models, two positions were defined along the symmetry axis using the “structure measurements” feature within UCSF Chimera. From the defined centroid positions, vectors along the symmetry axis of both oligomeric structures were derived. Tilt/twist angles were then calculated in the same way as described previously (1), and were compared to the tilt–twist distribution measured from cryo-ET data (*Tilt/twist analysis of CLPs*).

## Supplementary Material

Appendix 01 (PDF)

Movie S1. **3D visualisation of hexamer-hexamer tilt class series**. Hexamer-hexamer tilt class reconstructions, derived from classification of the single particle data set, are shown in series, from least tilted to most tilted (+6.3° → +24.9°). Unsharpened maps are shown to better illustrate the neighboring hexamers. The classified interaction partner is positioned towards the viewer.

Movie S2. **3D visualisation of hexamer-hexamer twist class series**. Hexamer-hexamer twist class reconstructions, derived from classification of the single particle data set, are shown in series, from negative twist to positive twist (-10.5° → +8.8°). Unsharpened maps are shown to better illustrate the neighboring hexamers. The classified interaction partner is positioned towards the viewer.

## Data Availability

Structures determined by electron microscopy are deposited in the Electron Microscopy Data Bank under accession codes EMD-16703 (50), EMD-16704 (51), EMD-16705 (52), EMD-16706 (53), EMD-16707 (54), EMD-16708 (55), EMD-16709 (56), EMD-16710 (57), EMD-16711 (58), and EMD-16712 (59). Corresponding molecular models are deposited in the Protein Data Bank under accession codes 8CKV (60), 8CKW (61), 8CKX (62), 8CKY (63), 8CKZ (64), 8CL0 (65), 8CL1 (66), 8CL2 (67), 8CL3 (68), and 8CL4 (69). Any additional information required to evaluate the conclusions of the paper is included in the paper.

## References

[1] S. Mattei, B. Glass, W. J. H. Hagen, H. G. Krausslich, J. A. G. Briggs, The structure and flexibility of conical HIV-1 capsids determined within intact virions. Science **354**, 1434–1437 (2016). 27980210 10.1126/science.aah4972

[2] B. K. Ganser, S. Li, V. Y. Klishko, J. T. Finch, W. I. Sundquist, Assembly and analysis of conical models for the HIV-1 core. Science **283**, 80–83 (1999). 9872746 10.1126/science.283.5398.80

[3] D. A. Jacques , HIV-1 uses dynamic capsid pores to import nucleotides and fuel encapsidated DNA synthesis. Nature **536**, 349–353 (2016). 27509857 10.1038/nature19098 PMC4998957

[4] X. Lahaye , The capsids of HIV-1 and HIV-2 determine immune detection of the viral cDNA by the innate sensor cGAS in dendritic cells. Immunity **39**, 1132–1142 (2013). 24269171 10.1016/j.immuni.2013.11.002

[5] J. Rasaiyaah , HIV-1 evades innate immune recognition through specific cofactor recruitment. Nature **503**, 402–405 (2013). 24196705 10.1038/nature12769 PMC3928559

[6] R. P. Sumner , DisruptingHIV-1 capsid formation causescGASsensing of viral DNA. Embo J. **39**, e103958 (2020). 32852081 10.15252/embj.2019103958 PMC7560218

[7] Q. Shen, C. X. Wu, C. Freniere, T. N. Tripler, Y. Xiong, Nuclear import of HIV-1. Viruses **13**, 2242 (2021). 34835048 10.3390/v13112242 PMC8619967

[8] J. Temple, T. N. Tripler, Q. Shen, Y. Xiong, A snapshot of HIV-1 capsid-host interactions. Curr. Res. Struct. Biol. **2**, 222–228 (2020). 34113849 10.1016/j.crstbi.2020.10.002 PMC8189282

[9] T. G. Muller, V. Zila, B. Muller, H. G. Krausslich, Nuclear capsid uncoating and reverse transcription of HIV-1. Annu. Rev. Virol. **9**, 261–284 (2022). 35704745 10.1146/annurev-virology-020922-110929

[10] R. A. Dick, D. L. Mallery, V. M. Vogt, L. C. James, IP6 regulation of HIV capsid assembly, stability, and uncoating. Viruses **10**, 640 (2018). 30445742 10.3390/v10110640 PMC6267275

[11] M. Obr, F. K. M. Schur, R. A. Dick, A structural perspective of the role of IP6 in immature and mature retroviral assembly. Viruses **13**, 1853 (2021). 34578434 10.3390/v13091853 PMC8473085

[12] N. Renner , A lysine ring in HIV capsid pores coordinates IP6 to drive mature capsid assembly. Plos Pathog. **17**, e1009164 (2021). 33524070 10.1371/journal.ppat.1009164 PMC7850482

[13] D. L. Mallery , IP6 is an HIV pocket factor that prevents capsid collapse and promotes DNA synthesis. Elife **7**, e35335 (2018). 29848441 10.7554/eLife.35335 PMC6039178

[14] T. Ni , Structure of native HIV-1 cores and their interactions with IP6 and CypA. Sci. Adv. **7**, eabj5715 (2021). 34797722 10.1126/sciadv.abj5715 PMC8604400

[15] P. Schommers , Changes in HIV-1 capsid stability induced by common cytotoxic-T-lymphocyte-driven viral sequence mutations. J. Virol. **90**, 7579–7586 (2016). 27279617 10.1128/JVI.00867-16 PMC4984629

[16] T. Ni , Intrinsic curvature of the HIV-1 CA hexamer underlies capsid topology and interaction with cyclophilin A. Nat. Struct. Mol. Biol. **27**, 855–862 (2020). 32747784 10.1038/s41594-020-0467-8 PMC8064030

[17] G. P. Zhao , Mature HIV-1 capsid structure by cryo-electron microscopy and all-atom molecular dynamics. Nature **497**, 643–646 (2013). 23719463 10.1038/nature12162 PMC3729984

[18] I. J. L. Byeon , Motions on the millisecond time scale and multiple conformations of HIV-1 CApsid protein: Implications for structural polymorphism of CA assemblies. J. Am. Chem. Soc. **134**, 6455–6466 (2012). 22428579 10.1021/ja300937v PMC3325613

[19] A. T. Gres , X-ray crystal structures of native HIV-1 capsid protein reveal conformational variability. Science **349**, 99–103 (2015). 26044298 10.1126/science.aaa5936 PMC4584149

[20] O. Pornillos, B. K. Ganser-Pornillos, M. Yeager, Atomic-level modelling of the HIV capsid. Nature **469**, 424–427 (2011). 21248851 10.1038/nature09640 PMC3075868

[21] T. R. Gamble , Crystal structure of human cyclophilin A bound to the amino-terminal domain of HIV-1 capsid. Cell **87**, 1285–1294 (1996). 8980234 10.1016/s0092-8674(00)81823-1

[22] K. Bichel , HIV-1 capsid undergoes coupled binding and isomerization by the nuclear pore protein NUP358. Retrovirology **10**, 81 (2013). 23902822 10.1186/1742-4690-10-81 PMC3750474

[23] F. Di Nunzio , Human nucleoporins promote HIV-1 docking at the nuclear pore, nuclear import and integration. Plos One **7**, e46037 (2012). 23049930 10.1371/journal.pone.0046037 PMC3457934

[24] A. Bhattacharya , Structural basis of HIV-1 capsid recognition by PF74 and CPSF6. Proc. Natl. Acad. Sci. U.S.A. **111**, 18625–18630 (2014). 25518861 10.1073/pnas.1419945112 PMC4284599

[25] A. J. Price , Host cofactors and pharmacologic ligands share an essential interface in HIV-1 capsid that is lost upon disassembly. Plos Pathogens **10**, e1004459 (2014). 25356722 10.1371/journal.ppat.1004459 PMC4214760

[26] S. V. Rebensburg , Sec24C is an HIV-1 host dependency factor crucial for virus replication. Nat. Microbiol. **6**, 435–444 (2021). 33649557 10.1038/s41564-021-00868-1 PMC8012256

[27] Y. Shinkai, M. Kuramochi, T. Miyafusa, New family members of FG repeat proteins and their unexplored roles during phase separation. Front. Dev. Biol. **9**, 708702 (2021). 10.3389/fcell.2021.708702 PMC8311347 34322491

[28] D. A. Bejarano , HIV-1 nuclear import in macrophages is regulated by CPSF6-capsid interactions at the nuclear pore complex. Elife **8**, e41800 (2019). 30672737 10.7554/eLife.41800 PMC6400501

[29] W. S. Blair , New small-molecule inhibitor class targeting human immunodeficiency virus type 1 virion maturation. Antimicrobial Agents and Chemotherapy **53**, 5080–5087 (2009). 19805571 10.1128/AAC.00759-09 PMC2786326

[30] K. Singh , GS-CA compounds: First-in-class HIV-1 capsid inhibitors covering multiple grounds. Front. Microbiol. **10**, 1227 (2019). 31312185 10.3389/fmicb.2019.01227 PMC6613529

[31] S. Segal-Maurer , Capsid inhibition with lenacapavir in multidrug-resistant HIV-1 infection. New England J. Med. **386**, 1793–1803 (2022). 35544387 10.1056/NEJMoa2115542

[32] R. A. Dick , Inositol phosphates are assembly co-factors for HIV-1. Nature **560**, 509–512 (2018). 30069050 10.1038/s41586-018-0396-4 PMC6242333

[33] C. M. Highland, A. Tan, C. L. Ricaña, J. A. G. Briggs, R. A. Dick, Structural insights into HIV-1 polyanion-dependent capsid lattice formation revealed by single particle cryo-EM. Proc. Natl. Acad. Sci. U.S.A. **120**, e2220545120 (2023). 10.1073/pnas.2220545120 PMC10160977 37094124

[34] R. T. Schirra, A molecular switch modulates assembly and host factor binding of the HIV-1 capsid. Nat. Struct. Mol. Biol. **33**, 383–390 (2023), 10.1038/s41594-022-00913-5. PMC10023569 36759579

[35] O. Pornillos , X-ray structures of the hexameric building block of the HIV capsid. Cell **137**, 1282–1292 (2009). 19523676 10.1016/j.cell.2009.04.063 PMC2840706

[36] Y. F. Chang, S. M. Wang, K. J. Huang, C. T. Wang, Mutations in capsid major homology region affect assembly and membrane affinity of HIV-1 gag. J. Mol. Biol. **370**, 585–597 (2007). 17532005 10.1016/j.jmb.2007.05.020

[37] U. K. von Schwedler, K. M. Stray, J. E. Garrus, W. I. Sundquist, Functional surfaces of the human immunodeficiency virus type 1 capsid protein. J. Virol. **77**, 5439–5450 (2003). 12692245 10.1128/JVI.77.9.5439-5450.2003 PMC153941

[38] V. Zila , Cone-shaped HIV-1 capsids are transported through intact nuclear pores. Cell **184**, 1032–1046 (2021). 33571428 10.1016/j.cell.2021.01.025 PMC7895898

[39] S. Q. Zheng , MotionCor2: Anisotropic correction of beam-induced motion for improved cryo-electron microscopy. Nat. Methods **14**, 331–332 (2017). 28250466 10.1038/nmeth.4193 PMC5494038

[40] D. Kimanius, L. Y. Dong, G. Sharov, T. Nakane, S. H. W. Scheres, New tools for automated cryo-EM single-particle analysis in RELION-4.0. Biochem. J. **478**, 4169–4185 (2021). 34783343 10.1042/BCJ20210708 PMC8786306

[41] T. Wagner , SPHIRE-crYOLO is a fast and accurate fully automated particle picker for cryo-EM. Commun. Biol. **2**, 218 (2019). 31240256 10.1038/s42003-019-0437-z PMC6584505

[42] A. Punjani, J. L. Rubinstein, D. J. Fleet, M. A. Brubaker, cryoSPARC: Algorithms for rapid unsupervised cryo-EM structure determination. Nat. Methods **14**, 290–296 (2017). 28165473 10.1038/nmeth.4169

[43] A. Punjani, H. W. Zhang, D. J. Fleet, Non-uniform refinement: Adaptive regularization improves single-particle cryo-EM reconstruction. Nat. Methods **17**, 1214–1221 (2020). 33257830 10.1038/s41592-020-00990-8

[44] E. F. Pettersen , UCSF chimera - A visualization system for exploratory research and analysis. J. Comput. Chem. **25**, 1605–1612 (2004). 15264254 10.1002/jcc.20084

[45] E. F. Pettersen , UCSF ChimeraX: Structure visualization for researchers, educators, and developers. Protein Sci. **30**, 70–82 (2021). 32881101 10.1002/pro.3943 PMC7737788

[46] T. I. Croll, ISOLDE: A physically realistic environment for model building into low-resolution electron-density maps. Acta Crystallogr. D Struct. Biol. **74**, 519–530 (2018). 29872003 10.1107/S2059798318002425 PMC6096486

[47] D. Liebschner , Macromolecular structure determination using X-rays, neutrons and electrons: Recent developments in Phenix. Acta Crystallogr. D Struct. Biol. **75**, 861–877 (2019). 31588918 10.1107/S2059798319011471 PMC6778852

[48] V. B. Chen , MolProbity: All-atom structure validation for macromolecular crystallography. Acta Crystallogr. D Struct. Biol. **66**, 12–21 (2010). 10.1107/S0907444909042073 PMC2803126 20057044

[49] A. Punjani, D. J. Fleet, 3D variability analysis: Resolving continuous flexibility and discrete heterogeneity from single particle cryo-EM. J. Struct. Biol. **213**, 107702 (2021). 33582281 10.1016/j.jsb.2021.107702

[50] J. C. V. Stacey, J. A. G. Briggs, HIV-1 mature capsid hexamer from CA-IP6 CLPs. Electron Microscopy Data Bank. https://www.ebi.ac.uk/emdb/16703. Deposited 16 February 2023.

[51] J. C. V. Stacey, J. A. G. Briggs, HIV-1 mature capsid pentamer from CA-IP6 CLPs. Electron Microscopy Data Bank. https://www.ebi.ac.uk/emdb/16704. Deposited 16 February 2023.

[52] J. C. V. Stacey, J. A. G. Briggs, HIV-1 mature capsid hexamer next to pentamer (type I) from CA-IP6 CLPs. Electron Microscopy Data Bank. https://www.ebi.ac.uk/emdb/16705. Deposited 16 February 2023.

[53] J. C. V. Stacey, J. A. G. Briggs, HIV-1 mature capsid hexamer from CA-IP6 CLPs, bound to Nup153 peptide. Electron Microscopy Data Bank. https://www.ebi.ac.uk/emdb/16706. Deposited 16 February 2023.

[54] J. C. V. Stacey, J. A. G. Briggs, HIV-1 mature capsid pentamer from CA-IP6 CLPs bound to Nup153 peptide. Electron Microscopy Data Bank. https://www.ebi.ac.uk/emdb/16707. Deposited 16 February 2023.

[55] J. C. V. Stacey, J. A. G. Briggs, HIV-1 mature capsid hexamer next to pentamer (type I) from CA-IP6 CLPs bound to Nup153 peptide. Electron Microscopy Data Bank. https://www.ebi.ac.uk/emdb/16708. Deposited 16 February 2023.

[56] J. C. V. Stacey, J. A. G. Briggs, HIV-1 mature capsid hexamer from CA-IP6 CLPs, bound to CPSF6 peptide. Electron Microscopy Data Bank. https://www.ebi.ac.uk/emdb/16709. Deposited 16 February 2023.

[57] J. C. V. Stacey, J. A. G. Briggs, HIV-1 mature capsid pentamer from CA-IP6 CLPs bound to CPSF6 peptide. Electron Microscopy Data Bank. https://www.ebi.ac.uk/emdb/16710. Deposited 16 February 2023.

[58] J. C. V. Stacey, J. A. G. Briggs, HIV-1 mature capsid hexamer from CA-IP6 CLPs, bound to Sec24C peptide. Electron Microscopy Data Bank. https://www.ebi.ac.uk/emdb/16711. Deposited 16 February 2023.

[59] J. C. V. Stacey, J. A. G. Briggs, HIV-1 mature capsid pentamer from CA-IP6 CLPs bound to Sec24C peptide. Electron Microscopy Data Bank. https://www.ebi.ac.uk/emdb/16712. Deposited 16 February 2023.

[60] J. C. V. Stacey, J. A. G Briggs, HIV-1 mature capsid hexamer from CA-IP6 CLPs. Protein Data Bank. ebi.ac.uk/pdbe/entry/pdb/8CKV. Deposited 16 February 2023.

[61] J. C. V. Stacey, J. A. G Briggs, HIV-1 mature capsid pentamer from CA-IP6 CLPs. Protein Data Bank. ebi.ac.uk/pdbe/entry/pdb/8CKW. Deposited 16 February 2023.

[62] J. C. V. Stacey, J. A. G Briggs, HIV-1 mature capsid hexamer next to pentamer (type I) from CA-IP6 CLPs. Protein Data Bank. ebi.ac.uk/pdbe/entry/pdb/8CKX. Deposited 16 February 2023.

[63] J. C. V. Stacey, J. A. G Briggs, HIV-1 mature capsid hexamer from CA-IP6 CLPs, bound to Nup153 peptide. Protein Data Bank. ebi.ac.uk/pdbe/entry/pdb/8CKY. Deposited 16 February 2023.

[64] J. C. V. Stacey, J. A. G Briggs, HIV-1 mature capsid pentamer from CA-IP6 CLPs bound to Nup153 peptide. Protein Data Bank. ebi.ac.uk/pdbe/entry/pdb/8CKZ. Deposited 16 February 2023.

[65] J. C. V. Stacey, J. A. G Briggs, HIV-1 mature capsid hexamer next to pentamer (type I) from CA-IP6 CLPs bound to Nup153 peptide. Protein Data Bank. ebi.ac.uk/pdbe/entry/pdb/8CL0. Deposited 16 February 2023.

[66] J. C. V. Stacey, J. A. G Briggs, HIV-1 mature capsid hexamer from CA-IP6 CLPs, bound to CPSF6 peptide. Protein Data Bank. ebi.ac.uk/pdbe/entry/pdb/8CL1. Deposited 16 February 2023.

[67] J. C. V. Stacey, J. A. G Briggs, HIV-1 mature capsid pentamer from CA-IP6 CLPs bound to CPSF6 peptide. Protein Data Bank. ebi.ac.uk/pdbe/entry/pdb/8CL2. Deposited 16 February 2023.

[68] J. C. V. Stacey, J. A. G Briggs, HIV-1 mature capsid hexamer from CA-IP6 CLPs, bound to Sec24C peptide. Protein Data Bank. ebi.ac.uk/pdbe/entry/pdb/8CL3. Deposited 16 February 2023.

[69] J. C. V. Stacey, J. A. G Briggs, HIV-1 mature capsid pentamer from CA-IP6 CLPs bound to Sec24C peptide. Protein Data Bank. ebi.ac.uk/pdbe/entry/pdb/8CL4. Deposited 16 February 2023.

