# Supplementary material for: Two structural switches in HIV-1 capsid regulate capsid curvature and host factor binding
Source: Proc Natl Acad Sci U S A. 2023 Apr 11;120(16):e2220557120. doi: 10.1073/pnas.2220557120 (PMC10120081; doi:10.1073/pnas.2220557120)
Supplement: Supplementary file 1 — Appendix 01 (PDF) [file pnas.2220557120.sapp.pdf]

PNAS

Supporting Information for
Two structural switches in HIV-1 capsid regulate capsid curvature and host factor binding

James C.V. Stacey[1,2], Aaron Tan[2,#], John M. Lu[2,§], Leo C. James[3], Robert A. Dick[4]*, John A.G. Briggs[1,2]*

John A.G. Briggs
Email: briggs@biochem.mpg.de

This PDF file includes:

Figures S1 to S12
Table S1
Legends for Movies S1 to S2
SI References

Other supporting materials for this manuscript include the following:

Movies S1 to S2

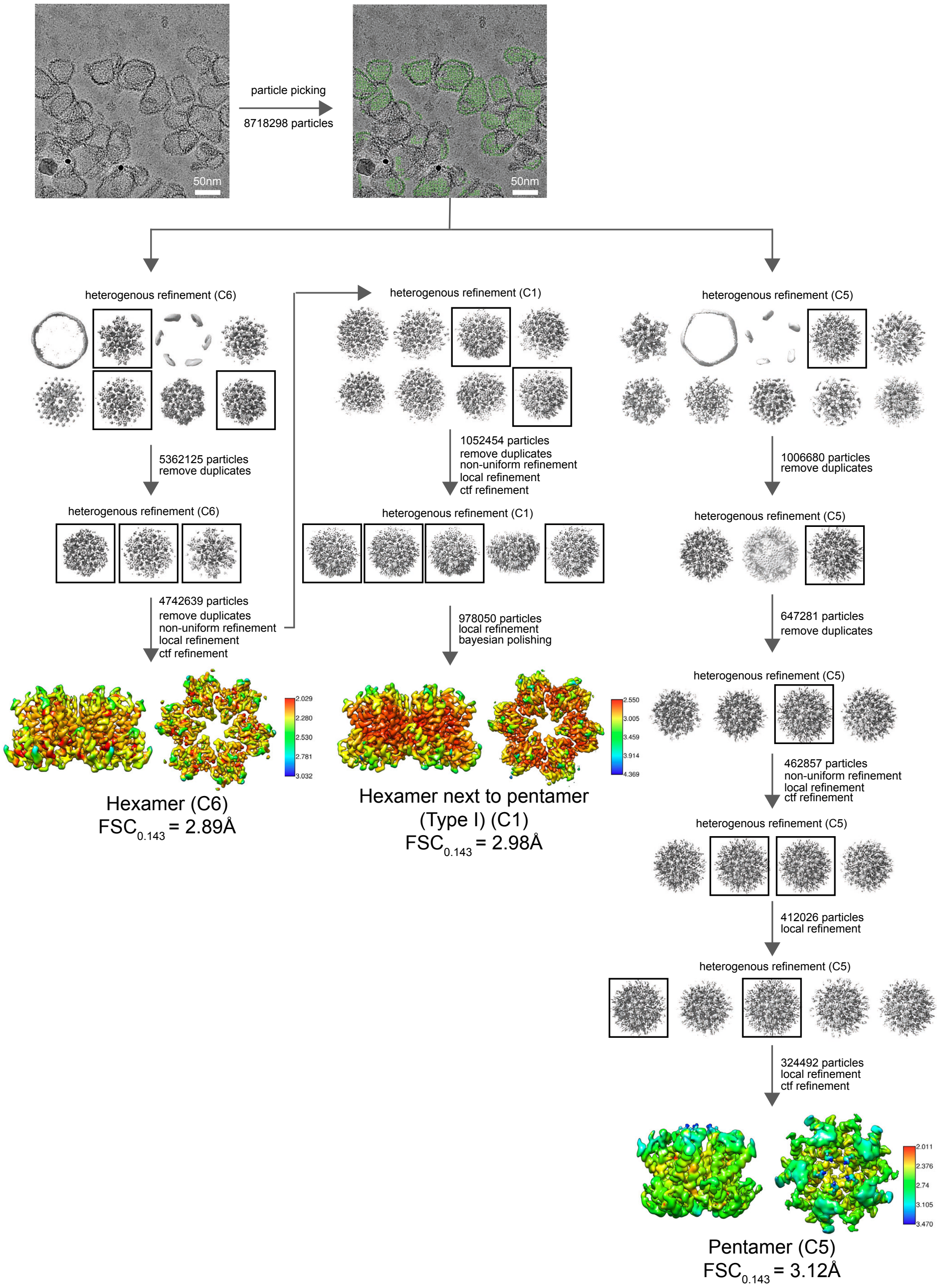

Fig. S1. Workflow for picking and processing of cryo-EM data – Apo CLPs

Pipeline for picking and processing of cryo-EM images of CLPs bound to no peptide (Apo). After automated picking of CLP surfaces in crYOLO, heterogenous refinement was used to sort hexamer and pentamer position as well to identify poor quality particles, which were discarded. Further classification was performed on the hexamer positions to identify hexamers directly adjacent to pentamers. Local resolution maps of all the final reconstructions, calculated in cryoSPARC, are shown.

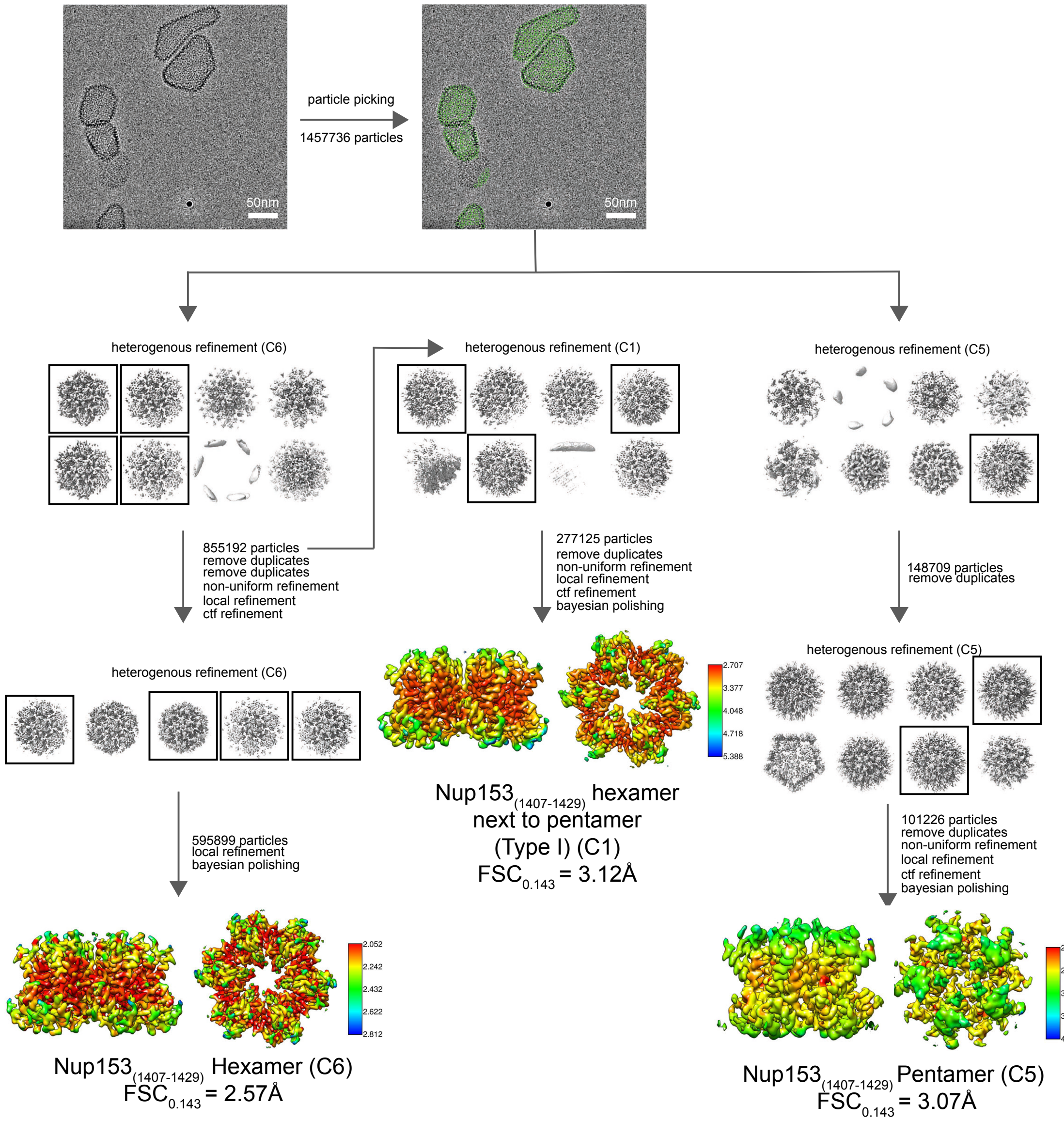

Fig. S2. Workflow for picking and processing of cryo-EM data: Nup153$_{(1407\text{-}1429)}$-bound CLPs

As in figure S1.

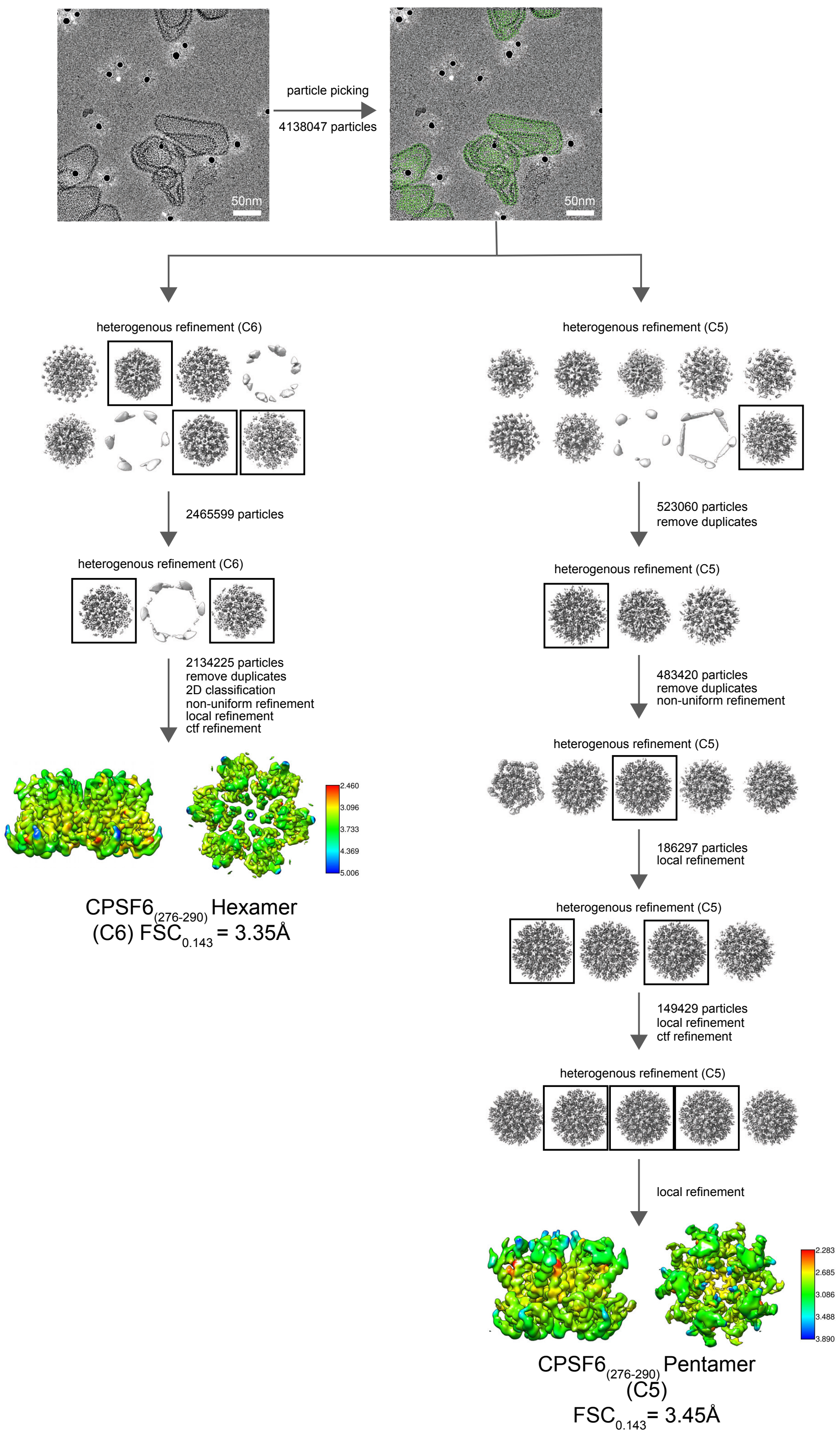

Fig. S3: Workflow for picking and processing of cryo-EM data: CPSF6$_{(276-290)}$-bound CLPs

As in figure S1 except that no further classification was performed to identify hexamer adjacent pentamer positions.

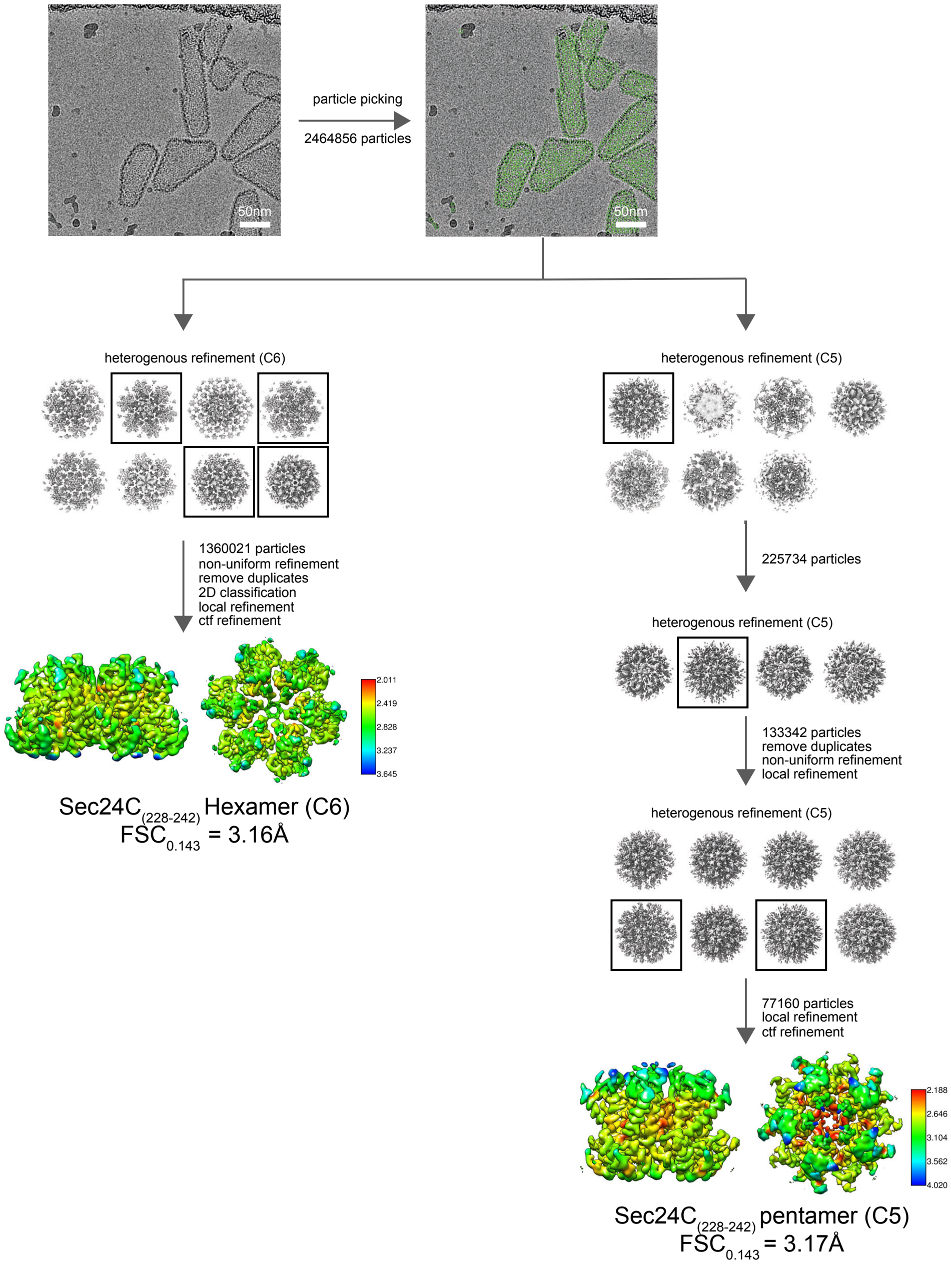

Fig. S4. Figure S4: Workflow for picking and processing of cryo-EM data: Sec24C$_{(228\text{-}242)}$-bound CLPs

As in figure S3.

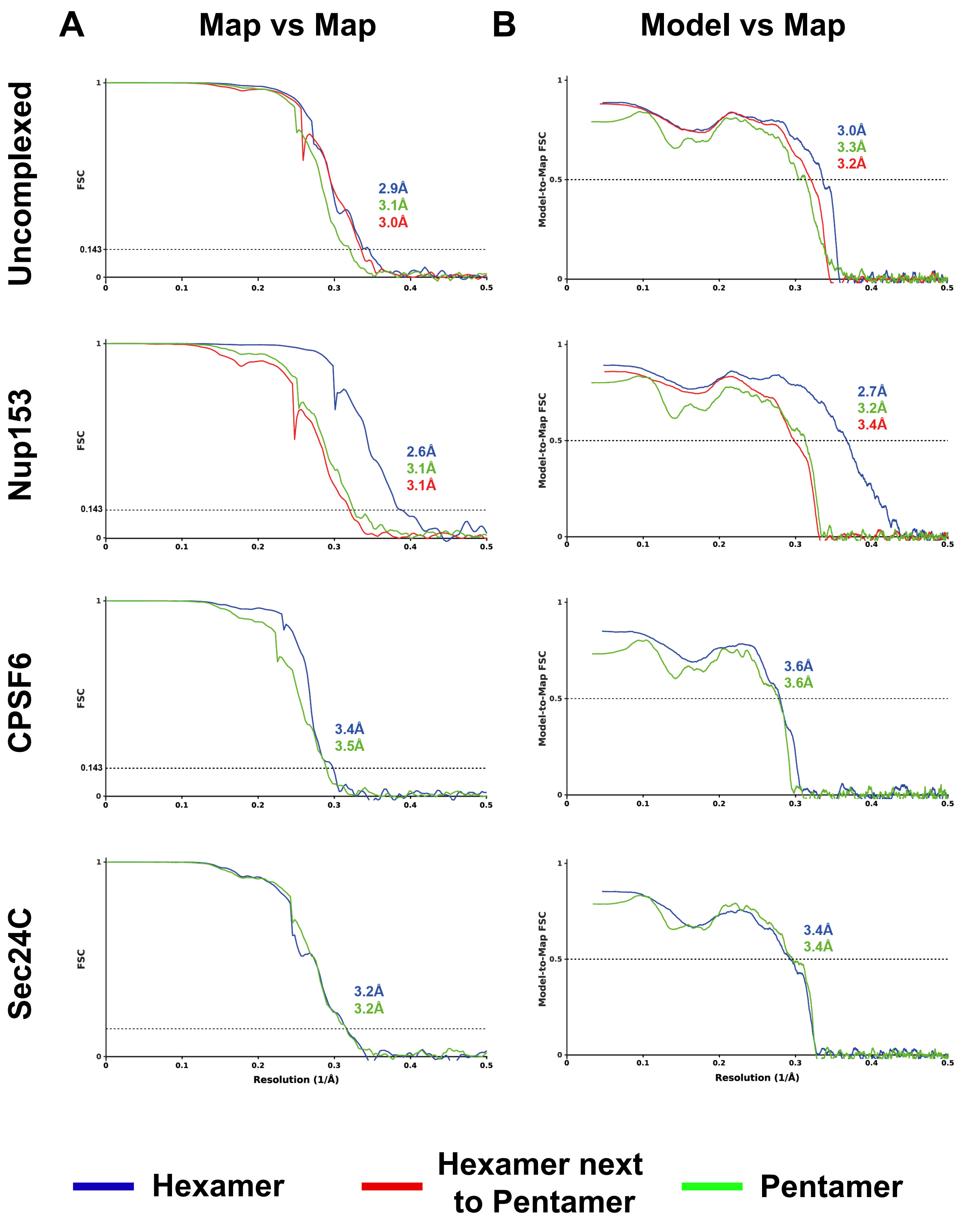

Fig. S5. Resolution assessment of cryo-EM structures

(A) Global resolution assessment of final reconstructions by Fourier shell correlation for each of the four datasets. **(B)** Fourier shell correlation between respective models and maps.

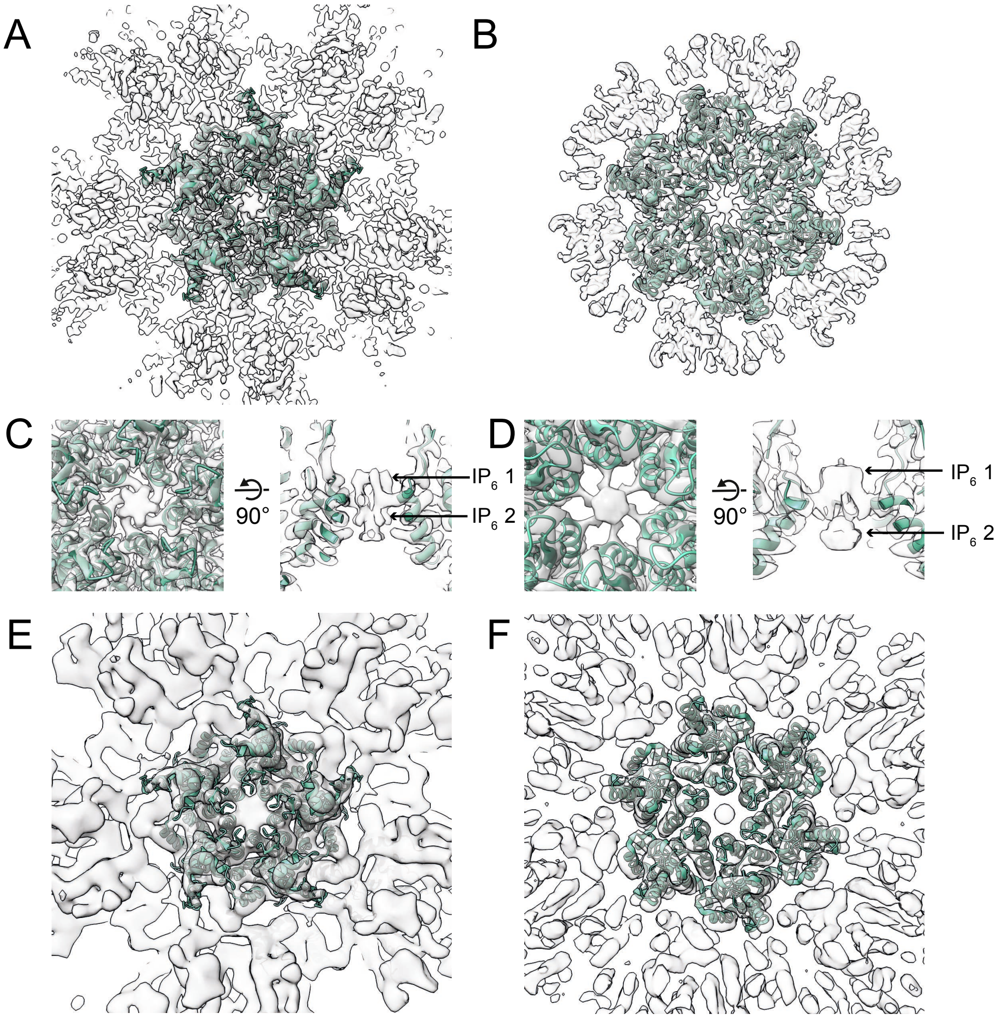

Fig. S6: Comparison of hexamer and pentamer structures with in-virus structures

(A) Model of the HIV-1 CA pentamer (green) determined from non-peptide bound CLPs is fit into the corresponding density (grey). **(B)** As in (A), for the hexamer. (C) Zoomed in view of the central pore region from the top (left) and side in cross-section (right), showing density that corresponds to two IP_6 molecules. **(D)** As in (C), for the hexamer, also showing two densities that correspond to IP_6. **(E)** Model from (A and C), fit into a reconstruction of the HIV-1 pentamer determined from intact virus particles by subtomogram averaging (EMD:3466) (1). **(F)** Model from (B and D), fit into a reconstruction of the HIV-1 hexamer determined from intact virus particles by subtomogram averaging (EMD-3465) (1).

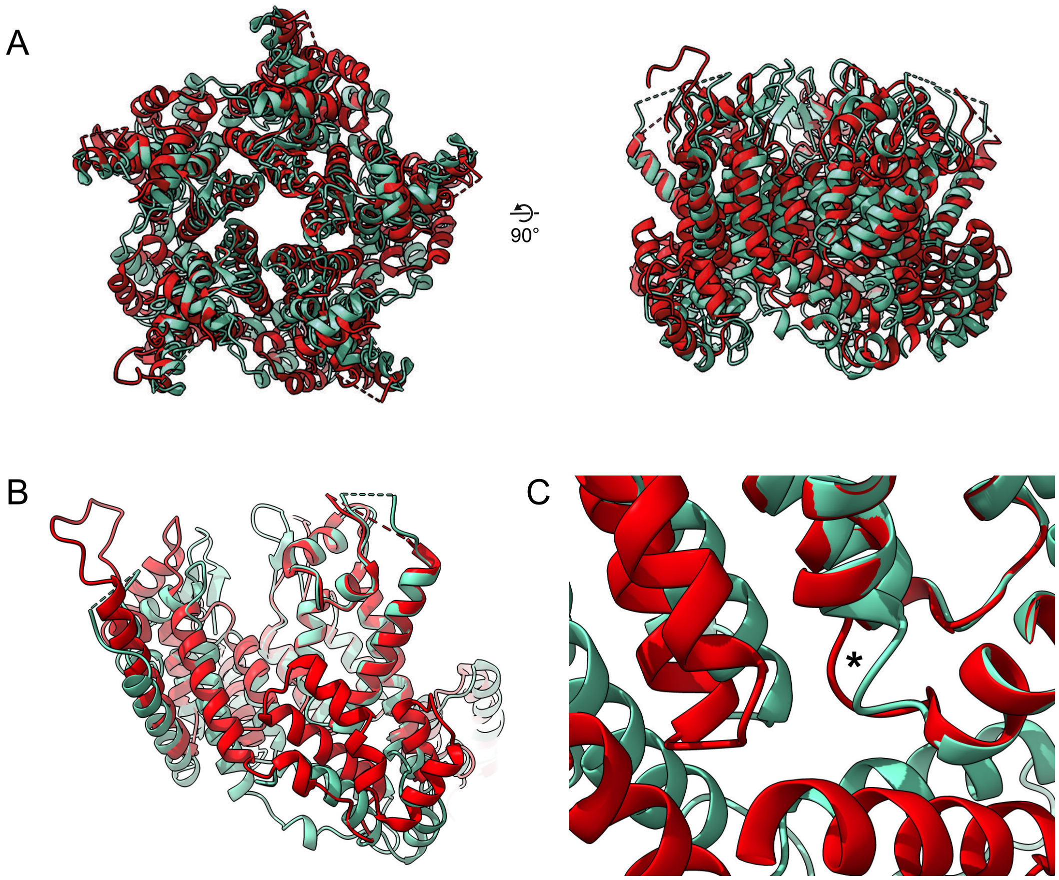

Fig. S7. Comparison of pentamer structure from CLPs with the pentamer crystal structure

(A) HIV-1 CA pentamer (green) determined from non-peptide bound CLPs superimposed with a previous pentamer structure engineered by the addition of disulfides and determined by X-ray crystallography (PDBID:3P05, red) (2), by fitting both into the pentamer EM density as in Figure S6A, shown as top view (left) and side view (right). **(B)** CA monomers from the CLP pentamer (green) and the crystallographic pentamer (red), aligned to the right-most NTD. The pentamer NTD-CTD interface in CLPs is different to that in the crystal structure. **(C)** Zoom in view of (B) at the pentamer-hexamer switch region (denoted by a * symbol). The switch conformation and the relative orientations between domains are different.

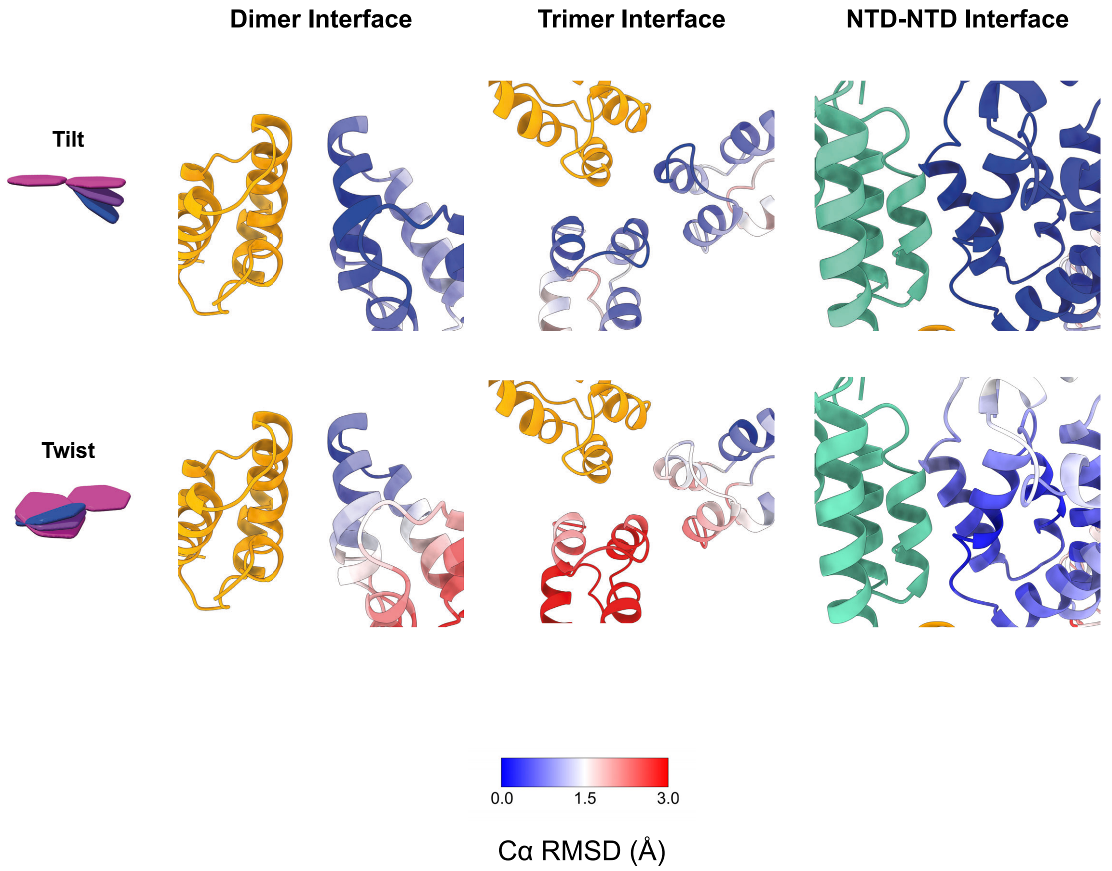

Fig. S8. Flexibility at CA hexamer-hexamer interfaces

The panels correspond to those in Figure 4C showing the dimeric and trimeric interfaces, as well as the region of helices 2 and 3 in the NTD. The models are colored according to the RMSD between structures obtained at low and high tilt and twist conformations from blue (0Å RMSD) to red (3Å RMSD).

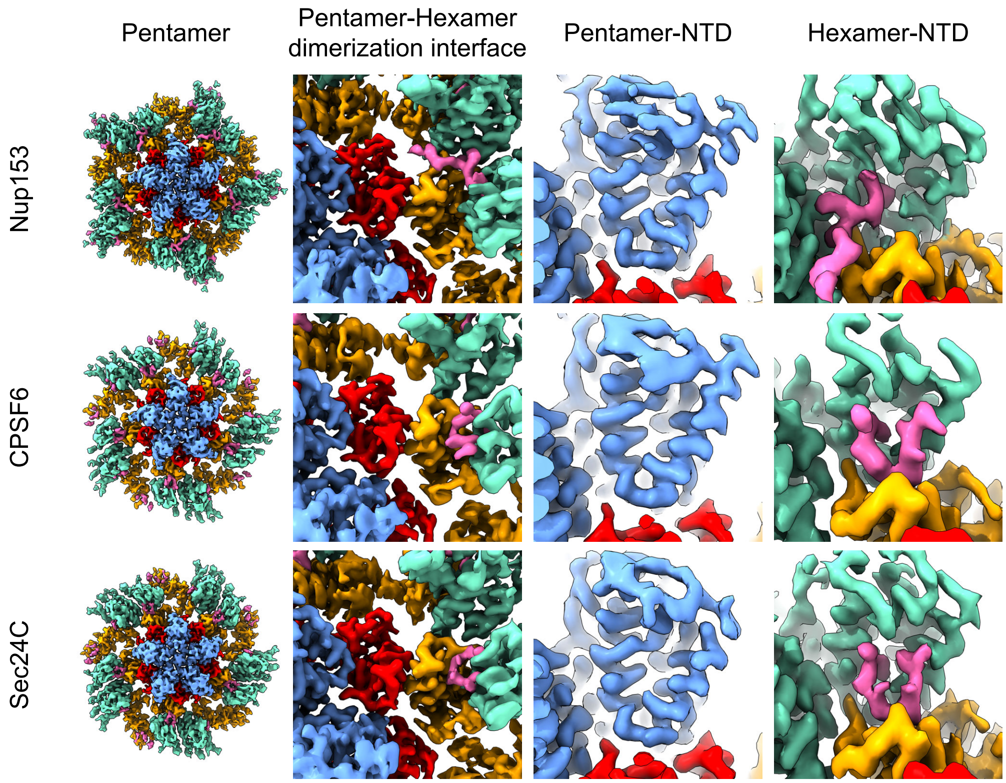

Fig. S9. Peptide density observed in hexamers adjacent to pentamers

Reconstructions of the HIV-1 CA pentamer determined from CLPs incubated with $Nup153_{(1407-1429)}$, $CPSF6_{(276-290)}$ or $Sec24C_{(228-242)}$. Density corresponding to the pentamer NTD and CTD are coloured blue and red respectively. Density corresponding to neighbouring hexamer molecule NTDs and CTDs are also resolved and are coloured green and orange respectively. Density corresponding to bound peptide within reconstructions is coloured pink. The second column shows a zoomed in view of the hexamer-pentamer dimerization interface for corresponding reconstructions. The third column shows a zoomed in view of corresponding pentamer NTD, showing no bound peptide in all three samples. The fourth column shows zoomed in view of the corresponding hexamer NTD, showing clear peptide density in all three samples.

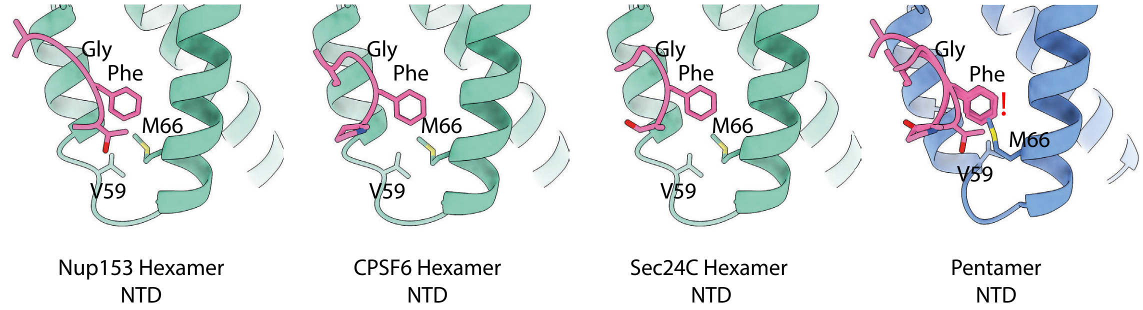

Fig. S10. The structure of the pentamer is incompatible with FG repeat motif binding

Models of the FG binding pocket from the CLPs incubated with $Nup153_{(1407-1429)}$, $CPSF6_{(276-290)}$ and $Sec24C_{(228-242)}$ (green), with the bound peptide (pink). On the right the three peptides are superimposed and are shown in the equivalent position in the CA pentamer (blue). The position of M66 in the pentamer is sterically incompatible with binding of the phenylalanine in the FG motif, the position of the clash is denoted by an exclamation mark.

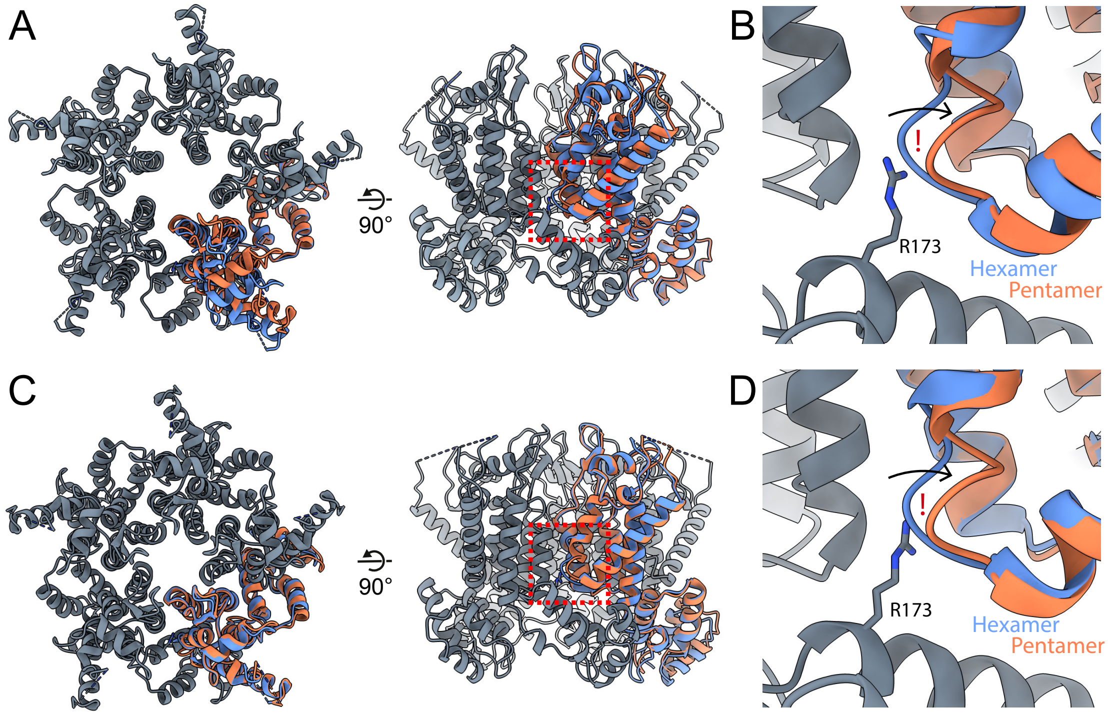

Fig. S11. The hexamer-pentamer switch changes to avoid steric clash with R173 in the pentamer

(A) A hypothetical pentamer constructed by rigidly fitting CA hexamer monomers into the pentamer reconstruction using only the CTD (grey, one monomer blue). A pentamer monomer is fit in the same way for comparison (orange). **(B)** Zoomed in view of the pentamer-hexamer switch region in the theoretical pentamer. R173 of the neighbouring CTD would clash with the pentamer-hexamer switch region if the switch was in the hexamer conformation. **(C)** The structure of the pentamer (grey, one monomer orange). A single monomer from the hexamer structure is aligned to the pentamer NTD (blue). **(D)** Zoomed in view of the pentamer-hexamer switch region in the pentamer. Again, R173 of the neighbouring CTD would clash with the pentamer-hexamer switch region if the switch was in the hexamer conformation.

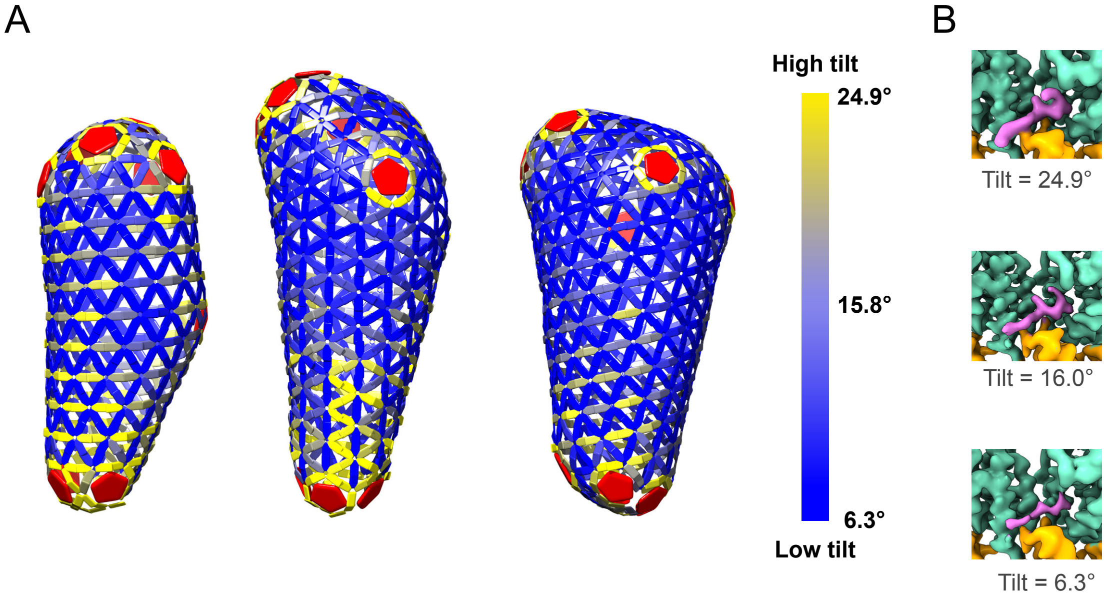

Fig. S12: Mapping of observed Nup153$_{(1407\text{-}1429)}$ binding preference onto 3D cores

(A) The hexamer-hexamer tilt angles are plotted on three CA-IP_6 cores derived from the tomography dataset. The tilt angle of the pair is indicated by the colour of a rectangle contacting its neighbour from yellow (high tilt) to blue (low tilt) and was calculated from the orientations of the hexamers. High-tilt regions are primarily, but not exclusively, located near pentamers. Pentamer positions are shown in red. Since Nup153$_{(1407\text{-}1429)}$ occupancy correlates with hexamer-hexamer tilt angle, this representation serves as a proxy for Nup153$_{(1407\text{-}1429)}$ binding preference across the core surface. **(B)** Isosurface volumes of Nup153$_{(1407\text{-}1429)}$ bound CA monomers engaging in low (+6.3°), medium (+16.0°) and high-tilt (+24.9°) lattice interactions, exactly as shown in Figure 5D.

Table S1. Cryo-EM data collection, refinement and validation statistics

	Apo hexamer EMD-16703 PDB 8CKV	Apo pentamer EMD-16704 PDB 8CKW	Apo hexamer next to pentamer (Type I) EMD-16705 PDB 8CKX	Nup153 hexamer EMD-16706 PDB 8CKY	Nup153 pentamer EMD-16707 PDB 8CKZ	Nup153 hexamer next to pentamer (Type I) EMD-16708 PDB 8CL0
**Data collection and processing**						
Magnification		130000			130000	
Voltage (kV)		300			300	
Electron exposure (e$^-$/Å^2)		40			40	
Defocus range (μm)		0.6-3.0			0.6-3.0	
Pixel size (Å)		0.93			0.93	
Movies (no.)		27252			4770	
Initial particle images (no.)		8718298			1457736	
Symmetry imposed	*C6*	*C5*	*C1*	*C6*	*C5*	*C1*
Final particle images (no.)	4309201	324357	975008	595899	101060	239458
Map resolution (Å)	2.89	3.12	2.98	2.57	3.07	3.12
FSC threshold	0.143	0.143	0.143	0.143	0.143	0.143
**Refinement**						
Initial model used	PDB 4XFX	PDB 4XFX	PDB 4XFX	PDB 4XFX PDB 5TSX	PDB 4XFX PDB 5TSX	PDB 4XFX PDB 5TSX
Model resolution (Å)	2.98	3.32	3.13	2.72	3.20	3.35
FSC threshold	0.5	0.5	0.5	0.5	0.5	0.5
Map sharpening *B* factor (Å^2)	-163.8	-120.0	-107.8	-60.0	-100.3	-90.8
Model composition						
Non-hydrogen atoms	1651	2779	9863	1731	2782	10395
Protein residues	212	357	1265	225	360	1356
B factors (Å^2)						
Protein	43.12	61.43	43.67	30.35	51.03	29.32
R.m.s. deviations						
Bond lengths (Å)	0.004	0.005	0.005	0.009	0.005	0.006
Bond angles (°)	0.823	0.886	1.205	0.874	0.912	0.898
**Validation**						
MolProbity score	0.84	0.81	0.74	0.61	0.85	1.14
Clashscore	1.22	1.08	0.76	0.29	1.26	3.50
Poor rotamers (%)	0	0	0.19	0.54	0	0.09
Ramachandran plot						
Favored (%)	99.04	100	99.03	99.54	98.86	99.77
Allowed (%)	0.96	0	0.97	0.46	1.14	0.23
Disallowed (%)	0	0	0	0	0	0

	Sec24C hexamer EMD-16711 PDB 8CL3	Sec24C pentamer EMD-16712 PDB 8CL4	CPSF6 hexamer EMD-16709 PDB 8CL1	CPSF6 pentamer EMD-16710 PDB 8CL2
**Data collection and processing**				
Magnification	130000		130000	
Voltage (kV)	300		300	
Electron exposure (e⁻/Å²)	40		40	
Defocus range (μm)	0.6-3.0		0.6-3.0	
Pixel size (Å)	0.93		0.93	
Movies (no.)	8784		21765	
Initial particle images (no.)	2464856		4138047	
Symmetry imposed	*C6*	*C5*	*C6*	*C5*
Final particle images (no.)	839656	77076	1035416	125966
Map resolution (Å)	3.16	3.17	3.35	3.45
FSC threshold	0.143	0.143	0.143	0.143
**Refinement**				
Initial model used	PDB 4XFX PDB 6PU1	PDB 4XFX PDB 6PU1	PDB 4XFX PDB 4U0A	PDB 4XFX PDB 4U0A
Model resolution (Å)	3.41	3.38	3.58	3.60
FSC threshold	0.5	0.5	0.5	0.5
Map sharpening *B* factor (Å²)	-133.0	-103.6	-155.8	-119.0
Model composition				
Non-hydrogen atoms	1712	2811	1749	2667
Protein residues	225	359	227	345
B factors (Å²)				
Protein	20.73	63.73	81.36	33.04
R.m.s. deviations				
Bond lengths (Å)	0.005	0.005	0.006	0.004
Bond angles (°)	0.978	0.919	1.196	0.841
**Validation**				
MolProbity score	0.77	1.18	1.01	0.64
Clashscore	0.89	3.91	2.31	0.841
Poor rotamers (%)	0	0	0	0
Ramachandran plot				
Favored (%)	99.09	99.15	100	100
Allowed (%)	0.91	0.85	0	0
Disallowed (%)	0	0	0	0

Movie S1 (separate file). 3D visualisation of hexamer-hexamer tilt class series.

Hexamer-hexamer tilt class reconstructions, derived from classification of the single particle data set, are shown in series, from least tilted to most tilted (+6.3° → +24.9°). Unsharpened maps are shown to better illustrate the neighboring hexamers. The classified interaction partner is positioned towards the viewer.

Movie S2 (separate file). 3D visualisation of hexamer-hexamer twist class series.

Hexamer-hexamer twist class reconstructions, derived from classification of the single particle data set, are shown in series, from negative twist to positive twist (-10.5° → +8.8°). Unsharpened maps are shown to better illustrate the neighboring hexamers. The classified interaction partner is positioned towards the viewer.

SI References

1. S. Mattei, B. Glass, W. J. H. Hagen, H. G. Krausslich, J. A. G. Briggs, The structure and flexibility of conical HIV-1 capsids determined within intact virions. Science **354**, 1434-1437 (2016).
2. O. Pornillos, B. K. Ganser-Pornillos, M. Yeager, Atomic-level modelling of the HIV capsid. *Nature* **469**, 424-427 (2011).
